# Anion-enhanced excited state charge separation in a spiro-locked N-heterocycle-fused push-pull zinc porphyrin†

**DOI:** 10.1039/d1sc00038a

**Published:** 2021-02-24

**Authors:** Mandeep K. Chahal, Anuradha Liyanage, Ajyal Z. Alsaleh, Paul A. Karr, Jonathan P. Hill, Francis D'Souza

**Affiliations:** International Centre for Materials Nanoarchitectonics (WPI-MANA), National Institute for Materials Science (NIMS) Namiki 1-1, Tsukuba Ibaraki 305-0044 Japan Jonathan.Hill@nims.go.jp; Department of Chemistry, University of North Texas 1155 Union Circle, #305070 Denton TX 76203-5017 USA Francis.dsouza@unt.edu; Department of Physical Sciences and Mathematics, Wayne State College 111 Main Street Wayne Nebraska 68787 USA

## Abstract

A new type of push–pull charge transfer complex, *viz.*, a spiro-locked N-heterocycle-fused zinc porphyrin, **ZnP-SQ**, is shown to undergo excited state charge separation, which is enhanced by axial F^−^ binding to the Zn center. In this push–pull design, the spiro-quinone group acts as a ‘lock’ promoting charge transfer interactions by constraining mutual coplanarity of the *meso*-phenol-substituted electron-rich Zn(ii) porphyrin and an electron deficient N-heterocycle, as revealed by electrochemical and computational studies. Spectroelectrochemical studies have been used to identify the spectra of charge separated states, and charge separation upon photoexcitation of **ZnP** has been unequivocally established by using transient absorption spectroscopic techniques covering wide spatial and temporal regions. Further, global target analysis of the transient data using GloTarAn software is used to obtain the lifetimes of different photochemical events and reveal that fluoride anion complexation stabilizes the charge separated state to an appreciable extent.

## Abstract 2

A new type of push–pull charge transfer complex, *viz.*, a spiro-locked N-heterocycle-fused zinc porphyrin, **ZnP-SQ**, is shown to undergo excited state charge separation, which is enhanced by axial F^−^ binding to the Zn center.

## Graphical abstract

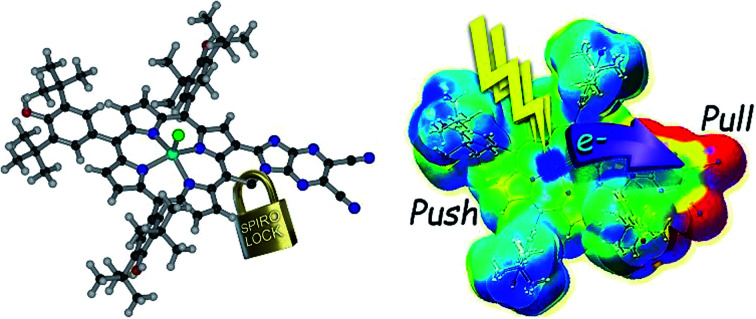

## Introduction

Molecules decorated with donor and acceptor moieties electronically conjugated through an appropriate intervening linking unit are often referred to as D–π–A molecules or ‘push–pull’ chromophores. The compounds illustrate how electronic structure can be controlled^1^ and are also of interest for applications including nonlinear optics,^2^ two photon absorption^3^ and dye-sensitization.^4^ Although several different π-electronic coupling linkers have been employed in these systems, the porphyrins^5^ are of special importance for this purpose because of their large synthetically flexible π-conjugated structures and excellent stabilities, the latter of which makes them attractive for molecular optics applications.^6^ Porphyrins present a wide range of highly developed synthetic strategies for incorporating the required donor (‘push’) and acceptor (‘pull’) groups, and many suitable candidates for applications are now available.^7–9^ The introduction of fused aromatic moieties such as benzo^10^ or naphtho^11^ groups also allows the porphyrin π-electronic system to be substantially expanded to tune the absorptive properties (wavelength, extinction) of the molecules for the required applications. Ring fusion also introduces the possibility of making highly complex π-electronic systems by conjoining different chromophores for multifunctionality.^12–15^

**Fig. 1:**
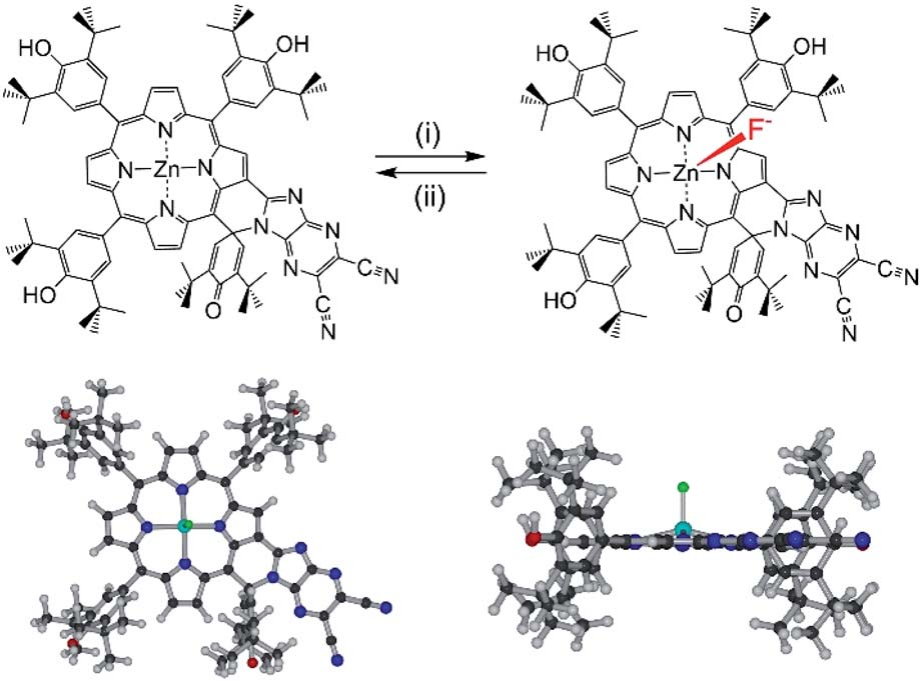
Top: chemical structure of spiro-locked N-heterocycle-fused zinc porphyrin, **ZnP-SQ**, prior to and following axial F^−^ binding; (i) tetra-*n*-butylammonium fluoride (TBAF), 1,2-dichlorobenzene (DCB); (ii) sodium tetrafluoroborate. Bottom: plan (left) and edge (right) views of the structure of **F−:ZnP-SQ** calculated at the B3LYP/6-31G* level.

In this work, we have considered a unique system containing two chromophores in which an electron rich phenol-substituted metalloporphyrin^16^ is fused with an electron deficient imidazo[4,5-*b*]pyrazine-5,6-dicarbonitrile unit^17–19^ leading to a push–pull type arrangement of the two chromophore, **ZnP-SQ** (Fig. 1). In this molecular design, the fused spiro-quinone group has the effect of constraining the 2-substituent locally coplanar with the porphyrin macrocycle enabling electronic push–pull interactions between the electron rich porphyrin unit and the highly electron deficient 2,3-dicyanopyrazine group. Excited state charge separation is probed in this system. Additionally, the effect of axial coordination of an anion, F^−^, in stabilizing the charge separation product^20^ was also probed.

## Results and discussion

### Synthesis of ZnP-SQ

**Scheme 1:**
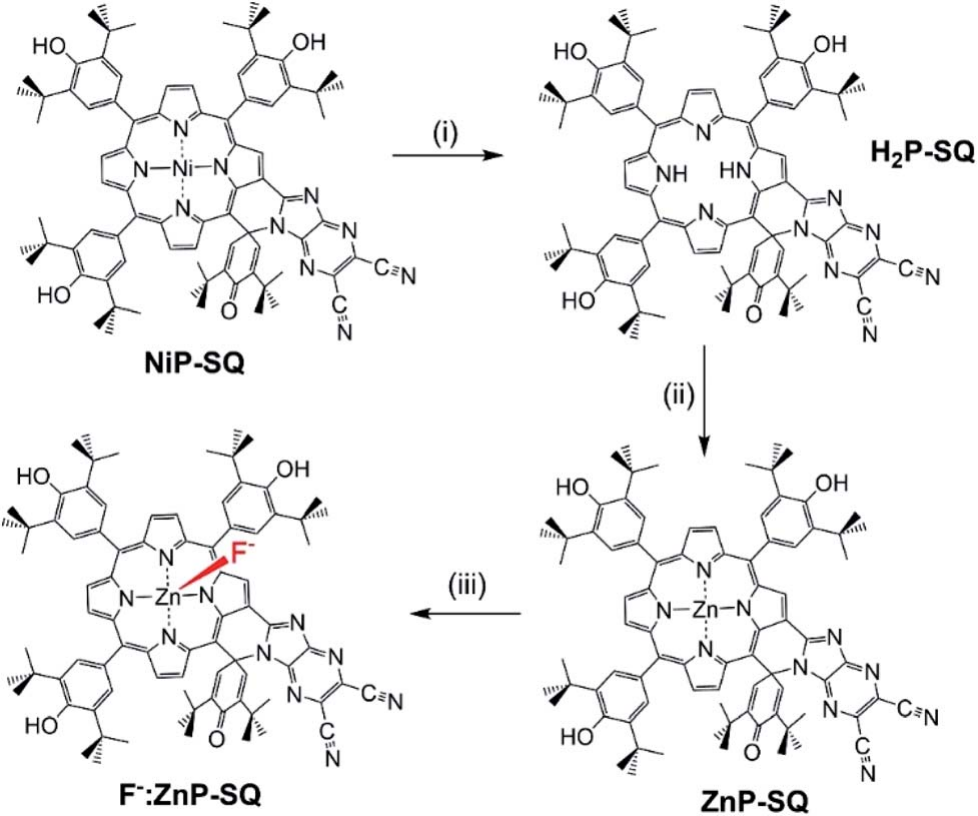
Transformation of **NiP-SQ** to **F−:ZnP-SQ**. (i) c. H_2_SO_4_. (ii) Zinc acetate/CHCl_3_/CH_3_OH. (iii) Teta-*n*-butylammonium fluoride.

Synthesis of **ZnP-SQ** was accomplished by exchange of Ni(ii) for Zn(ii) in the earlier reported **NiP-SQ**^18^ to minimize metal–ligand interactions but maintain the essential form of the molecule, as shown in Scheme 1. Demetallation of **NiP-SQ** was accomplished by its reaction with conc. aq. H_2_SO_4_ followed by neutralization to free-base porphyrin, and insertion of Zn(ii) by a known method (see ESI† for details of synthesis and spectral details). In order to monitor excited state charge transfer events in the present push–pull system, it was necessary to replace Ni(ii) with Zn(ii) in the porphyrin complex since the Ni(ii) complex is essentially non-fluorescent.

### Spectroscopic studies

**Fig. 2:**
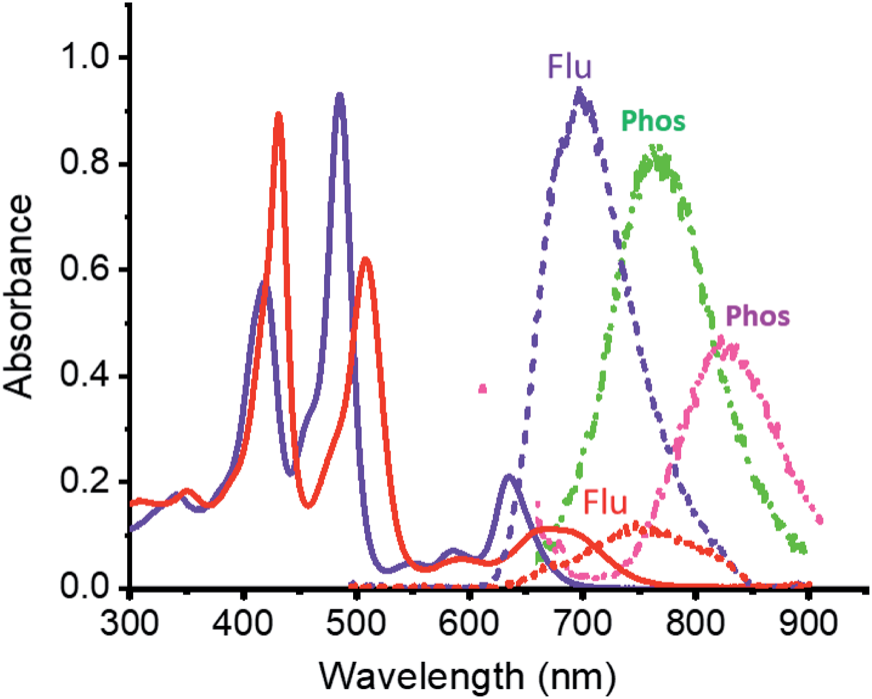
Absorption (solid line), fluorescence (short dash) and phosphorescence (dot dot dash, at 77 K) spectra of **ZnP-SQ** in dichlorobenzene (DCB) in the absence (purple) and presence of F^−^ (red for fluorescence and green (**ZnP-SQ**) and magenta (**F−:ZnP-SQ**) for phosphorescence). *λ*_EX_ = 485 nm.

Electronic absorption spectra of **ZnP-SQ** (Fig. 2) contain double absorption maxima with splitting due to reduced symmetry and bands located at 424 and 485 nm accompanied by ‘Q’ bands in the visible region at 588 and 636 nm. Fluorescence emission of **ZnP-SQ** (Fig. 2) occurs at 698 nm (*Φ*_f_ = 0.028 with respect to zinc tetraphenylporphyrin, **ZnTPP**, *Φ*_f_ = 0.03 in toluene^21^ while phosphorescence could also be observed with a maximum at 765 nm. From these spectral data, an energy of 1.89 eV (midpoint of 0,0 transitions of absorption and fluorescence) for the first excited state, and a singlet-triplet energy gap of 0.25 eV were determined. Fluorescence lifetimes determined by time correlated single photon counting (TCSPC) revealed a biexponential decay curve with lifetimes of 0.13 and 1.24 ns. The average lifetime of 1.24 ns is 0.56 ns lower than that observed for **ZnTPP** (∼1.80 ns) indicating that excited state events occur in **ZnP-SQ**.

Interactions of **ZnP-SQ** with fluoride anions (F^−^)^22^ were then investigated with spectral changes occurring during the course of F^−^ binding shown in Fig. S1a.† The complex **F−:ZnP-SQ** exhibits red-shifted absorption maxima at 432 and 510 nm, and Q bands in the visible region at 592 and 672 nm (Fig. 2). The binding constant, *K* (obtained from Benesi–Hildebrand plot^23^), was found to be 5.5 × 10^4^ M^−1^ (see Fig. S1a inset†). Binding of F^−^ also causes significant changes in fluorescence emission from **F−:ZnP-SQ** (Fig. S1b†); fluorescence maximum is red shifted to 750 nm (*Φ*_f_ = 0.026) with a reduced intensity; phosphorescence maximum also red shifted to 827 nm (Fig. 2). Biexponential decay was also observed for the **F−:ZnP-SQ** complex with lifetimes of 0.13 and 1.13 ns. Average lifetime was 1.13 ns which is shorter than that observed for **ZnP-SQ**. Interestingly, F^−^ binding could be reversed by addition of sodium tetraphenylborate leading to recovery of the original absorption and fluorescence spectra (Fig. S2†). Overall these data indicate that the presence of the highly electron deficient imidazo[4,5-*b*]pyrazine-5,6-dicarbonitrile moiety, whose fusion with porphyrin is enforced by the spiro-quinone entity, significantly diminishes the emission properties of **ZnP** in both **ZnP-SQ** and **F−:ZnP-SQ** suggesting the occurrence of intramolecular push–pull charge transfer events.^24^

### Computational, electrochemical and spectroelectrochemical studies

**Fig. 3:**
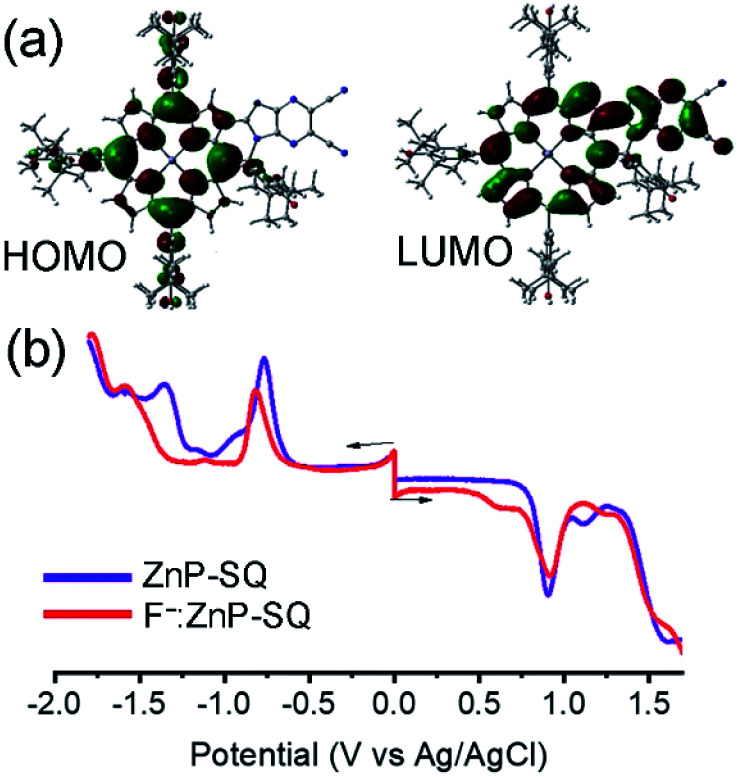
(a) HOMO and LUMO structures of **ZnP-SQ** on B3LYP/6-31G* optimized structure. (b) DPV of **ZnP-SQ** (purple) and **F−:ZnP-SQ** (red) in DCB containing 0.1 M (TBA)ClO_4_. Scan rate = 5 mV s^−1^, pulse width = 0.25 s, pulse height = 0.025 V.

In order to assess the push–pull activity in **ZnP-SQ**, additional DFT and electrochemical studies were performed. Fig. 3a shows HOMO and LUMO frontier orbitals of **ZnP-SQ** based on the B3LYP/6-31G* optimized structure.^25^ The HOMO is located largely on the porphyrin π-system with substantial contributions on the three phenolic substituents, while the LUMO also lies on the porphyrin π-system but with a large conjugated contribution on the electron deficient fused imidazo[4,5-*b*]pyrazine-5,6-dicarbonitrile unit. The location of the orbital coefficients indicates the relative electron richness and deficiency in **ZnP-SQ**, and this is supported by the molecular electrostatic potential map (MEP, Fig. S3†) where push–pull structure is obvious (see figure in TOC). Cyclic voltammetry studies performed in DCB revealed quasireversible redox processes (up to three reductions and two oxidations) with some reversibility at high scan rates and at low temperature (Fig. S4†).

**Fig. 4:**
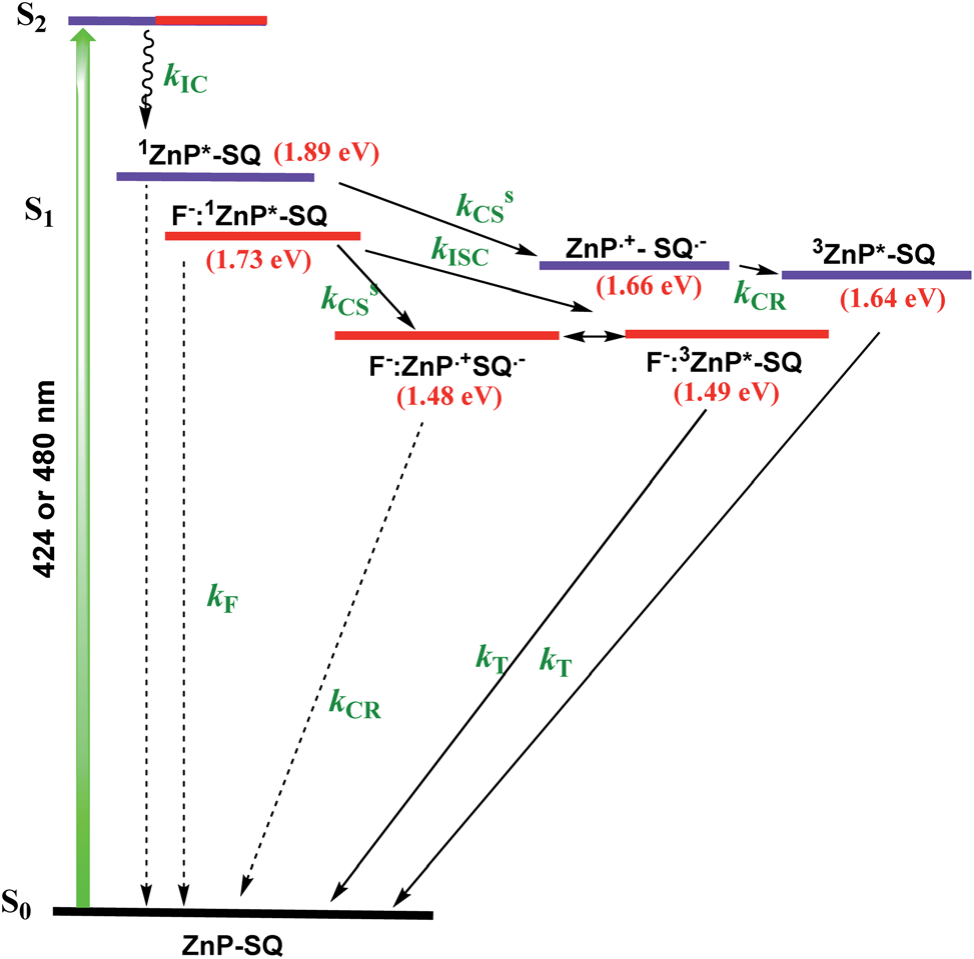
Energy level diagram depicting excited state charge separation and recombination upon Soret band excitation in **ZnP-SQ** (purple) and **F−:ZnP-SQ** (red). Abbreviations: F = fluorescence, IC = internal conversion, ISC = intersystem crossing, CS = charge separation, CR = charge recombination, S = singlet state and T = triplet state. Solid arrow = major path, dashed arrow = minor path.

Differential pulse voltammetry (DPV, Fig. 3b) was used to obtain the redox potentials. First oxidation and first reduction of **ZnP-SQ** are located at 0.91 and −0.74 V *vs.* Ag/AgCl with these potentials being cathodically-shifted to 0.65 and −0.83 V in **F−:ZnP-SQ** due to additional negative charge contributed by F^−^ coordination. The electrochemical HOMO–LUMO gaps are 1.66 and 1.48 V for **ZnP-SQ** and **F−:ZnP-SQ**, respectively. For comparison, respective *E*_0,0_ values of 1.98 and 1.73 eV were calculated from spectroscopic data. Depression of the HOMO–LUMO gaps obtained from electrochemical data suggest the possibility of excited state charge separation in these push–pull systems, as summarized in the energy level shown in Fig. 4. When considering Fig. 4, it is important to note that the energy of the charge separated state, **ZnP˙+-SQ˙−**, calculated according to the Rehm–Weller approach,^26,27^ is about 0.02 eV higher than the level of 3ZnP*-SQ while for the complex **F−:ZnP˙+-SQ˙−**, the CS state and F−:3ZnP*-SQ are isoenergetic. Under these conditions, the charge separated state can populate the triplet state during relaxation to the ground state.

Spectroelectrochemistry is a powerful tool for the spectroscopic characterization of the products of electron transfer during any oxidation and reduction processes. For strongly coupled push–pull systems based on an extensive π-electronic structure of the type studied here, both oxidation and reduction are known to perturb the electronic structure resulting in new optical transitions,^1^ and the present system is no exception. Spectral changes observed during first oxidation and first reduction of **ZnP-SQ** and **F−:ZnP-SQ** are shown in Fig. S5.† Oxidation of **ZnP-SQ** leads to new peaks at 546, 688, and a broad peak at 940 nm due to its radical cation form, while reduction is accompanied by new peaks at 508 and 742 nm corresponding to a radical anion. Similar trends were observed for **F−:ZnP-SQ** (Fig. S5†). To identify the products of charge separation during the subsequent transient absorption studies, spectroelectrochemical data was used to obtain differential absorption spectrum of the charge separated species, *viz.*, **ZnP˙+-SQ˙−** and **F−:ZnP˙+-SQ˙−**. In this procedure, spectra of oxidized and reduced species were digitally summed then subtracted from the spectrum of the neutral compound. Spectra corresponding to the charge separated states, **ZnP˙+-SQ˙−** and **F−:ZnP˙+-SQ˙−**, are shown in Fig. S5† (bottom panel). The most important features of these spectra are the depleted intensity of the neutral **ZnP-SQ** at 435, 485, 587 and 636 nm, and the positive peaks at 516, 562 and 740 corresponding to **ZnP˙+-SQ˙−**. Similar trends were observed for the spectrum deduced for **F−:ZnP˙+-SQ˙−**.

### Transient absorption studies

**Fig. 5:**
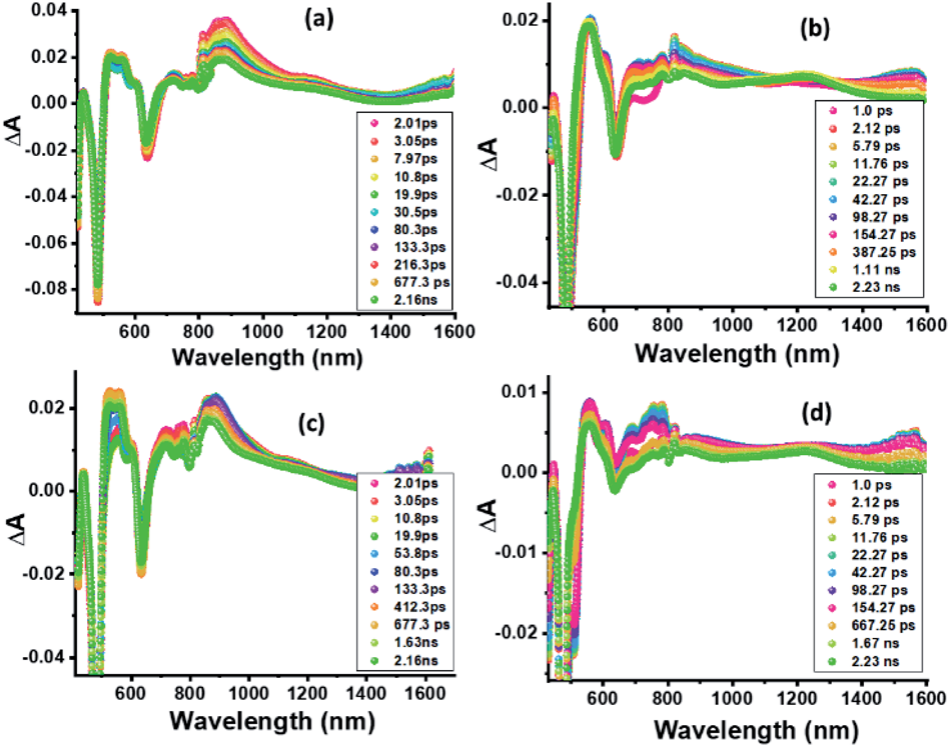
Fs-TA spectra at the indicated delay times of (a and c) **ZnP-SQ** and (b and d) **F−:ZnP-SQ** at the excitation wavelength of 424 (a and b) and 480 nm (c and d) in DCB.

In order to characterize spectrally the predicted charge separation products, femtosecond transient absorption (fs-TA) studies were performed. The samples were excited at both absorption maxima in the UV region (split Soret). Fig. 5 shows the fs-TA spectra at the indicated delay times for **ZnP-SQ** and **F−:ZnP-SQ** with excitation at 424 and 480 nm. It may be pointed here that in the presence of low concentrations of F^−^,^28^ the **F−:ZnP-SQ** complex is expected in an equilibrium mixture of bound and unbound complexes. Immediately following excitation, the instantaneously formed S_2_ state of **ZnP** relaxes on a subpicosecond timescale to populate the S_1_ state. For **ZnP-SQ**, 1ZnP*-SQ exhibits excited state absorption (ESA) peaks at 528, 563, 607 and 887 nm with depleted peaks at 425, 483 and 638 nm due to ground state bleach (GSB) also emerging. Decay and recovery of ESA and GSB peaks is accompanied by the new peaks expected for the **ZnP˙+-SQ˙−** charge separation product at 518, 562 and 730 nm. At longer delay times, peaks corresponding to **ZnP˙+-SQ˙−** were attenuated with concurrent appearance of new peaks around the 520, 850 and 1200 nm.

**Fig. 6:**
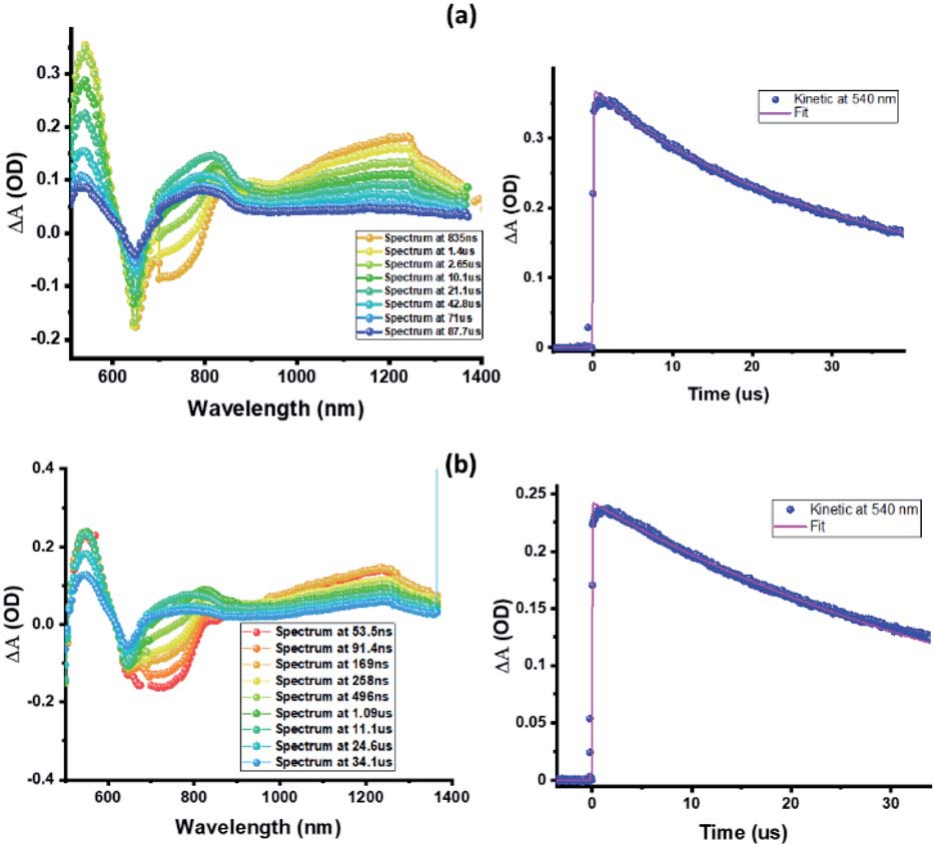
Nanosecond transient absorption spectra at the indicated delay times of (a) **ZnP-SQ** and (b) **F−:ZnP-SQ** at the excitation wavelength of 480 nm in DCB.

Nanosecond transient absorption (ns-TA) spectral studies (Fig. 6) revealed that the spectral features observed at longer delay times are due to formation of 3ZnP*-SQ since peaks due to a triplet–triplet transition at the expected wavelengths were observed. These peaks could be eliminated by purging solutions with O_2_. For 3ZnP*-SQ, a biexponential decay was observed with lifetimes of 17.8 and 87.0 μs, while for F−:3ZnP*-SQ, a monoexponential decay with a lifetime of 48.2 μs was observed. Overall, it is possible to establish that excited state charge separation occurs leading to **ZnP˙+-SQ˙−** and **F−:ZnP˙+-SQ˙−**, with subsequent recombination to the **ZnP** triplet state.

**Fig. 7:**
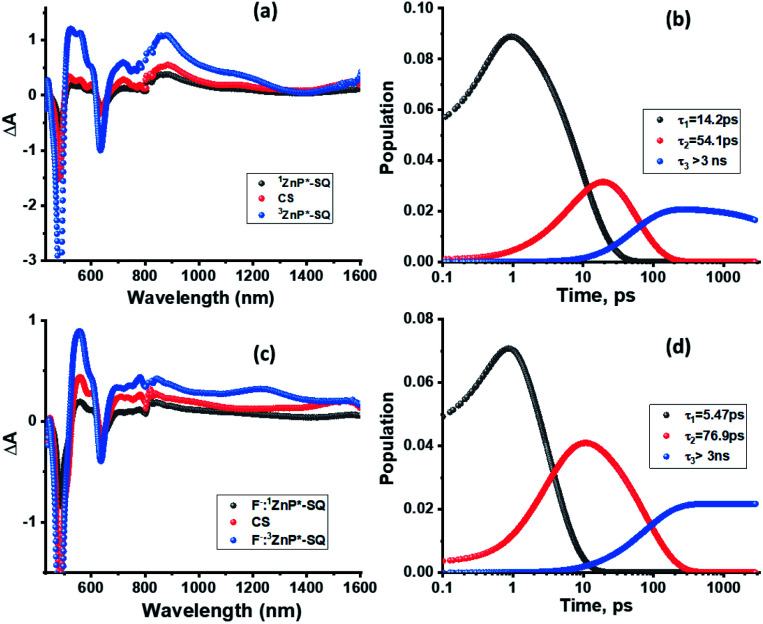
Species associated spectra (a and c) and population kinetics (b and d) of **ZnP-SQ** (a and b) and **F−:ZnP-SQ** (c and d) at the excitation wavelength of 424 nm.

Next, in order to determine the kinetics of charge separation events in these systems, the spectral data was analyzed using the GloTaRan software suite.^29,30^ A three-step kinetic model, *viz.*, 1ZnP* formation, charge separation leading to the formation of radical ion-pair, and 3ZnP* formation during the process of charge recombination were considered in the data fitting. Fig. 7 shows the ‘species associated spectra’ (SAS) and population kinetics for **ZnP-SQ** and **F−:ZnP-SQ** at the excitation wavelength of 424 nm. Results obtained for 480 nm excitation are shown in Fig. S6.† The population kinetic values in these plots represent the average lifetime of the corresponding transient species.

From the data shown in Fig. 7 and S6,† it is clear that 1ZnP* formation is accelerated to some extent upon axial ligation of F^−^ to Zn(ii) (14.2 ps and 5.46 ps respectively for **ZnP-SQ** and **F−:ZnP-SQ** with excitation at 424 nm, and 36.8 ps and 11.4 ps respectively for **ZnP-SQ** and **F−:ZnP-SQ** with excitation at 480 nm). Interestingly, persistence of the charge separated state was improved to some extent in the presence of F^−^ independent of the excitation wavelength (54.1 ps and 76.9 ps respectively for **ZnP-SQ** and **F−:ZnP-SQ** with excitation at 424 nm, and 191.6 ps and 206.0 ps respectively for **ZnP-SQ** and **F−:ZnP-SQ** with excitation at 480 nm). The third component, attributed to 3ZnP* formation, has lifetimes greater than 3 ns, the maximum delay time of the fs-TA setup, which agrees well with its longer lifetime as discussed earlier for the ns-TA spectral studies.

It should also be mentioned that although excited state charge transfer and charge separation in porphyrin push–pull systems have been reported,^7–12^ the present system containing a directly fused spiro-locked N-heterocycle is unique in the sense that the pull group is directly attached to the electron rich zinc porphyrin, which acts as the push group. We have also been successful in demonstrating the effect of axial coordinated F^−^ for modulation of the charge transfer events.

## Conclusions

In summary, the push–pull charge transfer molecule **ZnP-SQ**, a novel spiro-locked N-heterocycle-fused zinc porphyrin, undergoes excited state charge separation, which is enhanced by axial F^−^ binding to the Zn centre. The spiro-quinone group acts as a ‘lock’ which, in this case, promotes charge transfer type interactions by constraining mutual coplanarity of the *meso*-phenol-substituted electron-rich Zn(ii) porphyrin and an electron deficient N-heterocycle, as revealed by electrochemical and computational studies. Spectroelectrochemical studies were used to identify the spectra of charge separated states, and charge separation upon photoexcitation of **ZnP** was unequivocally established by using fs-TA spectroscopy. GloTarAn^29,30^ software was used to obtain the lifetimes of different photochemical events and revealed that fluoride anion complexation enhances the stability of the charge separated state to an appreciable extent. Further studies of intramolecular charge transfer in similar push–pull molecules based on the spiro-quinone lock involving other substituents are underway in our laboratories.

## Note added after first publication

This article replaces the version published on 24 Feb 2021, which contained errors in the first sentence of reference 26.

## Conflicts of interest

The authors declare no conflicts of interest.

## Supplementary Material

SC-012-D1SC00038A-s001

SC-012-D1SC00038A-s002
