# Supplementary material for: Anion-enhanced excited state charge separation in a spiro-locked N-heterocycle-fused push-pull zinc porphyrin
Source: Chem Sci. 2021 Feb 24;12(13):4925–30. doi: 10.1039/d1sc00038a (PMC8179616; doi:10.1039/d1sc00038a)
Supplement: SC-012-D1SC00038A-s001 [file SC-012-D1SC00038A-s001.pdf]

Electronic Supplementary Material (ESI) for Chemical Science.
This journal is © The Royal Society of Chemistry 2021

Electronic Supplementary Information

Anion-enhanced Excited State Charge Separation in a Spiro-locked N-Heterocycle-fused Push-Pull Zinc Porphyrin

Mandeep K. Chahal,[a] Anuradha Liyanage,[b] Ajyal Z. Alsaleh,[b] Paul A. Karr,[c] Jonathan P. Hill*[a] and Francis D'Souza*[b]

International Center for Materials Nanoarchitectonics (WPI-MANA), National Institute for Materials Science (NIMS), Namiki 1-1, Tsukuba, Ibaraki, 305-0044, Japan. E-mail: Jonathan.Hill@nims.go.jp

Department of Chemistry, University of North Texas, 1155 Union Circle, #305070, Denton, TX 76203-5017, United States; E-mail: Francis.dsouza@unt.edu.

Department of Physical Sciences and Mathematics, Wayne State College, 111 Main Street, Wayne, Nebraska, 68787, USA.

Contents

1.0 General Experimental....S2

2.0 Synthesis S4

3.0 Additional analytical data....S7

4.0 Chemical Analytical Spectra S15

1.0 General Experimental

Reagents and dehydrated solvents (in septum-sealed bottles) used for syntheses and spectroscopic measurements were obtained from Tokyo Kasei Chemical Co., Wako Chemical Co. or Aldrich Chemical Co. and were used as received without further purification. Electronic absorption spectra were measured using a JASCO V-570 UV/Vis/NIR spectrophotometer. ATR-FTIR spectra were obtained using a Thermo-Nicolet 760X FTIR spectrophotometer equipped with a SMART-iTX ATR accessory. ^{1}H-NMR spectra were obtained using JEOL AL300BX or JEOL AL400SSS spectrometers operating respectively at 300 and 400 MHz with tetramethylsilane as an internal standard. Proton decoupled ^{13}C-NMR spectra were obtained using JEOL AL300BX or JEOL AL400SSS spectrometers operating respectively at 76 and 101 MHz with tetramethylsilane as an internal standard. Data processing was performed using Delta version 5.0.5.1, Always JNM-AL version 6.2 or MestReNova 6.0.2 software packages. ^{1}H NMR chemical shifts (δ) are reported in ppm relative to TMS for $CDCl_3$ (δ 0.00) or the residual solvent peak in other solvents. ^{13}C NMR chemical shifts (δ) are reported in ppm relative to the solvent reported. Coupling constants (*J*) are expressed in Hertz (Hz), shift multiplicities are reported as singlet (s), doublet (d), triplet (t), quartet (q), double doublet (dd), multiplet (m) and broad singlet (bs). High resolution ESI-MS mass spectra were measured using a Thermo Scientific Q-Exactive Plus instrument. Tetrakis(3,5-di-*t*-butyl-4-hydroxyphenyl)porphinatonickel(II) ([T(DtBHP)P]Ni) and **NiP-SQ** were prepared according to a literature method.[S1]

Electronic absorption spectroscopy was carried out using a Shimadzu Model 2550 double-monochromator UV−visible spectrophotometer. Fluorescence emission and phosphorescence emission spectra were measured by using a Horiba Yvon Nanolog with time-correlated single-photon counting and nanoLED excitation sources. Orthogonal detection method was used to record fluorescence emission and a pulse xenon lamp was used to record phosphorescence. Differential pulse and cyclic voltammograms were recorded using an EG&G PARSTAT electrochemical analyzer and a three-electrode system with a platinum button working electrode, platinum wire as a counter electrode, and a Ag/AgCl reference electrode. Ferrocene/ferrocenium redox couple was used as an internal standard. All solutions were purged using nitrogen gas prior to electrochemical and spectral measurements.

Femtosecond transient absorption spectroscopy experiments were performed using an ultrafast femtosecond laser source (Libra) by Coherent incorporating a diode-pumped, mode locked Ti:sapphire laser (Vitesse) and a diode-pumped intracavity doubled Nd:YLF laser (Evolution) to generate a compressed laser output of 1.45 W. For optical detection, a Helios transient absorption spectrometer coupled with a femtosecond harmonics generator, both provided by Ultrafast Systems LLC, was used. The sources for the pump and probe pulses were derived from the fundamental output of Libra (Compressed output 1.45 W, pulse width 100 fs) at a repetition rate of 1 kHz; 95% of the fundamental output of the laser was introduced into a TOPAS-Prime-OPA system with a 290−2600 nm tuning range from Altos Photonics Inc., (Bozeman, MT), while the rest of the output was used for generation of a white light continuum. Kinetic traces at appropriate wavelengths were assembled from the time-resolved spectral data. Data analysis was performed using Surface Xplorer software supplied by Ultrafast Systems. All measurements were conducted in degassed solutions at 298 K. The estimated error in the reported rate constants is ±10%.

The nanosecond transient absorption measurements was performed using a laser flash photolysis instrumental setup composed of an Opolette HE 355 LD pumped using a high-energy Nd:YAG laser with second and third harmonics OPO (tuning range 410−2200 nm, pulse repetition rate 20 Hz, pulse length 7 ns) with laser power of 1.0−3 mJ/pulse. For spectral measurements, a Proteus UV−vis−NIR flash photolysis spectrometer (Ultrafast Systems, Sarasota, FL) with a fiberoptic delivering white light as probe and either a fast rise Si photodiode detector (covering 200−1000 nm range) or a InGaAs photodiode detector (covering 900−1600 nm range) was used. The output from the photodiodes and a photomultiplier tube was recorded using a digitizing Tektronix oscilloscope. Data analysis was performed using Surface Xplorer software from Ultrafast Systems.

2.0 Synthesis

H_2P-SQ

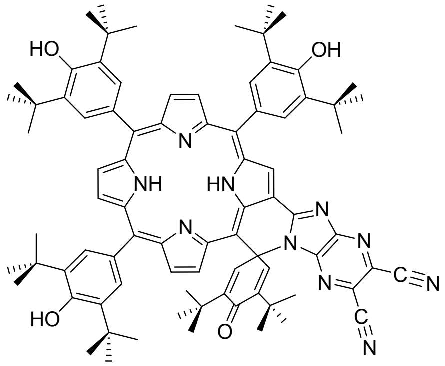

NiP-SQ[S1] (60 mg, 0.044 mmol) was dissolved in $CHCl_3$ (8 mL), conc. H_2SO_4 (0.1 mL) was added dropwise, and the resulting mixture stirred vigorously at 0 °C for 1 hour. Distilled water (40 mL) was then added and the organic layer was separated and neutralized using 25% aqueous ammonia solution. The organic layer was then dried over anhydrous Na_2SO_4 followed by filtration and removal of solvents under reduced pressure. The resulting residue was purified by column chromatography on silica using $CHCl_3$ as an eluent. Product containing fractions were combined and the solvent removed under reduced pressure yielding **H_2P-SQ** (40 mg, 70%).

UV/Vis: λ / nm (CH_2Cl_2 / M^{-1} cm^{-1}): 339 (28600), 416 (73000), 479 (141800), 547 (17700), 588 (7100), 628 (7100) and 686 (13500) nm. ^1H NMR (400 MHz, $CDCl_3$): δ = 10.07 (s, 1H), 9.56 (d, J = 5.5 Hz, 1H), 9.05 (s, 2H), 9.03 (d, J = 5.5 Hz, 1H), 8.83 (d, J = 4.6 Hz, 1H), 8.75 (d, J = 4.6 Hz, 1H), 8.04 (s, 2H), 8.00 (s, 2H), 7.98 (s, 2H), 7.21 (s, 2H), 5.69 (s, 1H), 5.61 (s, 1H), 5.60 (s, 1H), 1.66 (s, 18H), 1.63 (s, 36H), 1.37 (s, 18H), -2.35 - -2.18 (m, 2H) ppm. ^{13}C NMR (101 MHz, $CDCl_3$): δ = 185.30, 157.96, 157.13, 156.98, 154.55, 154.24, 154.17, 153.74, 152.60, 147.82, 144.95, 141.55, 140.22, 139.66, 138.98, 138.89, 136.91, 135.89, 135.80, 135.55, 134.68, 134.62, 134.34, 133.05, 132.56, 132.27, 132.24, 132.15, 131.94, 131.14, 130.49, 129.64, 129.03, 128.96, 125.31, 124.70, 124.57, 124.51, 123.44, 114.29, 113.77, 108.07, 65.62, 35.95, 34.81, 30.84, 29.92, 29.57 ppm. FT-IR(ATR): ν = 3630.2 (m), 3303.1 (w), 2954.9 (m), 2869.9 (m), 2237.4 (w), 1725.8 (w), 1668.1 (w), 1626.8 (s), 1573.8 (w), 1546.5 (w), 1523.5 (w), 1465.8 (m), 1432.0 (s), 1402.8 (s), 1363.2 (w), 1341.3 (w), 1321.4 (m), 1235.3 (s), 1153.6 (w), 1136.3 (w), 1119.1 (s), 1059.9 (w), 1024.9 (w), 982.1 (m), 921.1 (m), 888.6 (w), 850.2 (w), 832.5 (w), 803.8 (m), 794.1 (w), 785.5 (w), 772.5 (w), 756.6 (w), 725.4 (w), 714.0 (w), 672.6 (w), 654.0 (w), 630.9 (w) cm^{-1}. HRMS (ESI-MS, CH_2Cl_2): calculated for $[C_{83}H_{93}O_4N_{10}]^+$ = 1293.7376, found: = 1293.7349.

ZnP-SQ

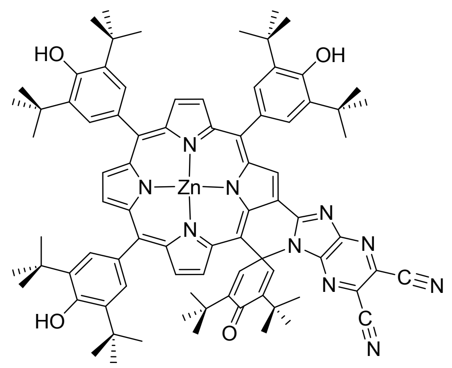

Chemical Formula: $C_{83}H_{90}N_{10}O_4Zn$

H_2P-SQ (50 mg, 0.039 mmol) was dissolved in $CHCl_3$ (10 mL). $Zn(OAc)_2{\cdot}2H_2O$ (10 equiv.) dissolved in methanol (2 mL) was then added and the resulting mixture refluxed for 40 m. The reaction mixture was then cooled to room temperature, washed with water and the organic fraction dried over anhydrous Na_2SO_4. The crude product was purified by column chromatography on silica using $CHCl_3$ as an eluent yielding **ZnP-SQ** (46 mg, 88%).

UV/Vis: λ / nm (CH_2Cl_2 / M^{-1} cm^{-1}): 339 (28800), 414 (92200), 480 (134400), 546 (6900), 583 (10600) and 632 (29700). ^{1}H NMR (400 MHz, $CDCl_3$): δ = 10.08 (s, 1H), 9.61 (d, J = 5.0 Hz, 1H), 9.00 - 9.05 (m, 4H), 8.95 (d, J = 5.0 Hz, 1H), 8.06 (s, 2H), 8.00 (s, 2H), 7.99 (s, 2H), 7.35 (s, 2H), 5.68 (s, 1H), 5.60 (s, 1H), 5.59 (s, 1H), 1.64-1.68 (m, 54H), 1.40 (s, 18H) ppm. ^{13}C NMR (101 MHz, $CDCl_3$): δ = 185.46, 158.39, 154.47, 154.13, 154.10, 153.26, 153.21, 152.72, 152.33, 152.02, 151.12, 150.09, 148.67, 147.23, 141.94, 141.86, 140.29, 134.81, 134.59, 134.53, 134.39, 134.20, 134.06, 133.83, 133.62, 133.40, 133.14, 132.97, 132.10, 132.05, 131.90, 129.88, 128.74, 126.11, 125.48, 124.54, 124.12, 123.91, 122.39, 114.23, 113.76, 109.60, 66.38, 36.03, 34.94, 34.76, 30.99, 29.69 ppm. FT-IR(ATR): ν = 3632.1 (m, OH(str)), 2955.0 (m, CH(str)), 2914.3 (m, CH(str)), 2869.9 (m, CH(str)), 2235.6 (w, C≡N(str)), 1667.9 (w), 1624.3 (s), 1572.0 (w), 1534.2 (w), 1480.2 (m), 1429.5 (s), 1408.5 (s), 1363.5 (m), 1339.1 (w), 1320.8 (m), 1234.7 (m), 1217.1 (m), 1196.2 (w), 1145.1 (m), 1121.0 (m), 1074.8 (w), 1026.1 (w), 1006.1 (m), 956.7 (w), 937.1 (m), 888.7 (w), 853.5 (w), 815.8 (w), 800.1 (m), 783.2 (w), 772.2 (w), 756.9 (w), 716.3 (m), 676.0 (w), 654.8 (w), 633.8 (w) cm^{-1}. HRMS (ESI-MS, CH_2Cl_2); calculated for $[C_{83}H_{90}O_4N_{10}Zn]^+$ = 1354.6432, found: = 1354.6425.

<u>F^-:ZnP-SQ</u>

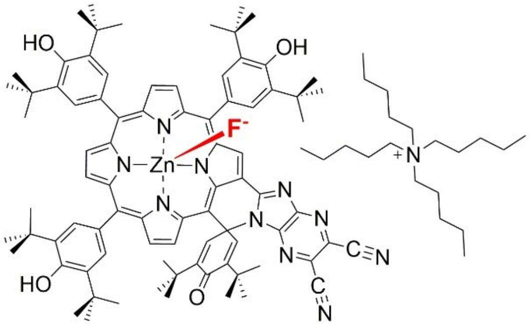

Chemical Formula: $[C_{83}H_{90}N_{10}O_4Zn{:}F^-][TnBA^+]$

ZnP-SQ was dissolved in chloroform and titrated with TnBAF until its ^{1}H-NMR spectrum was invariant indicating saturation of binding at the Zn(II) site (approx. 2 eq; see Fig. S7). This is consistent also with the electronic spectroscopy measurements indicating strong interaction of F^- with Zn(II) and the binding constant. No deprotonation of the phenol moieties was detected. The solution of **F-ZnP-SQ** was then used for purposes of the characterization of **F-ZnP-SQ** as detailed below. The infrared spectrum was collected by drying the solution on the ATR window. ESI-TOF-MS were also collected from the solution.

UV/Vis: λ / nm (CH_2Cl_2 / M^{-1} cm^{-1}): 350 (7800), 429 (37200), 508 (21900), 587 (2300) and 677 (4300). ^{1}H NMR (400 MHz, $CDCl_3$, 2 eq. TnBAF): δ = 9.92 (s, 1H), 9.38 (d, J = 5.0 Hz, 1H), 8.75-8.83 (m, 5H), 7.98-8.04 (m, 6H), 5.48-4.97 (-OH protons), 1.61-1.64 (m, 54H), 1.34 (s, 18H) ppm. ^{13}C NMR (101 MHz, $CDCl_3$, 2 eq. TnBAF): δ = 186.05, 159.70, 153.76, 153.49, 153.37, 153.28, 153.22, 153.12, 152.22, 151.74, 151.50, 150.04, 148.77, 146.29, 142.27, 141.96, 141.19, 134.66, 134.38, 134.30, 133.98, 133.93, 133.69, 133.22, 132.85, 132.75, 132.51, 132.19, 131.81, 131.25, 129.43, 129.12, 128.34, 125.11, 124.18, 123.77, 122.82, 121.31, 114.84, 114.26, 107.85, 58.27, 35.84, 34.83, 30.99, 29.65 ppm. FT-IR(ATR): ν = 3635.4 (w, OH(str)), 2956.3 (m, CH(str)), 2872.5 (m, CH(str)), 2235.9 (w, C≡N(str)), 1719.5 (w, C=O(str)), 1666.6 (w), 1621.5 (s), 1532.6 (w), 1509.7 (w), 1475.7 (m), 1428.3 (m), 1412.0 (m), 1389.4 (w), 1358.2 (m), 1332.1 (m), 1322.8 (m), 1276.9 (w), 1248.1 (w), 1232.5 (w), 1215.6 (m), 1143.8 (m), 1129.1 (m), 1065.6 (w), 1026.2 (w), 1000.0 (m), 952.6 (w), 935.4 (m), 907.9 (w), 853.2 (w), 813.5 (w), 799.5 (m), 779.8 (w), 728.7 (s), 717.9 (s), 693.6 (m), 675.6 (m), 640.2 (w) cm^{-1}. HRMS (ESI-TOF-MS, CH_2Cl_2); calculated for $[C_{83}H_{90}FN_{10}O_4Zn]^-$ = 1373.642, found: = 1373.622.

3.0 Additional analytical data

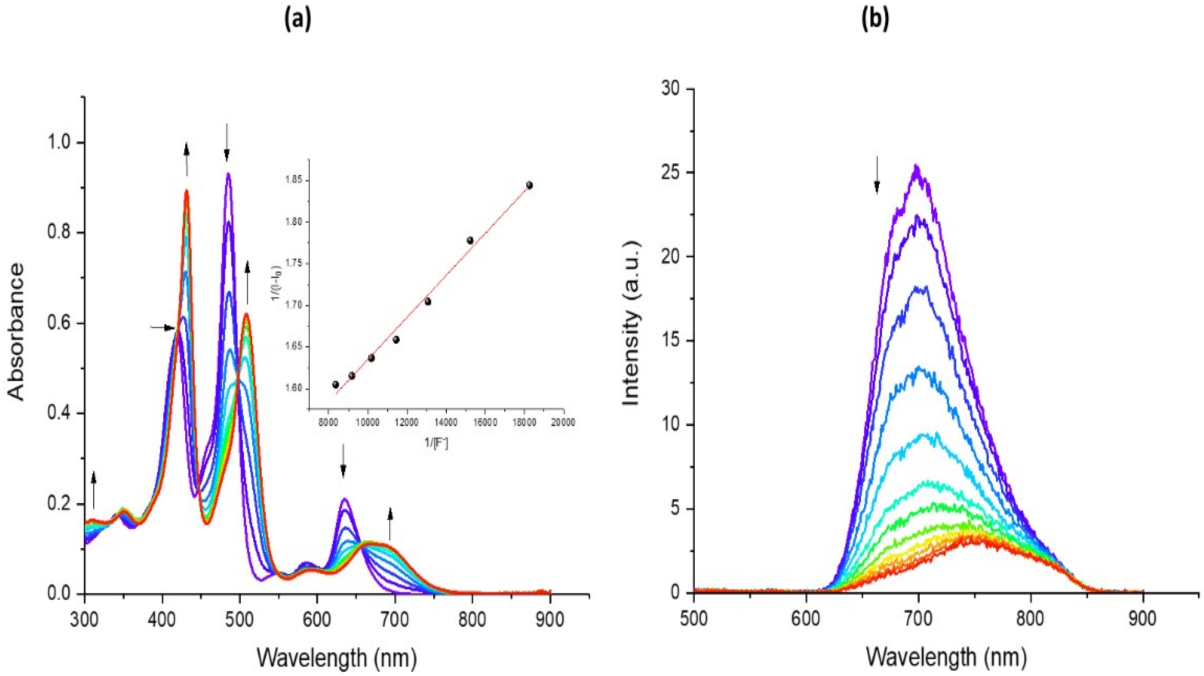

Figure S1. (a) Absorption and (b) fluorescence (excitation = 485 nm) spectral changes observed during addition of TBAF (2 equivalents each) to a solution of **ZnP-SQ** (5.5 x 10^{-6} M) in DCB. Figure inset shows Benesi-Hildebrand plot constructed to evaluate the binding constant.

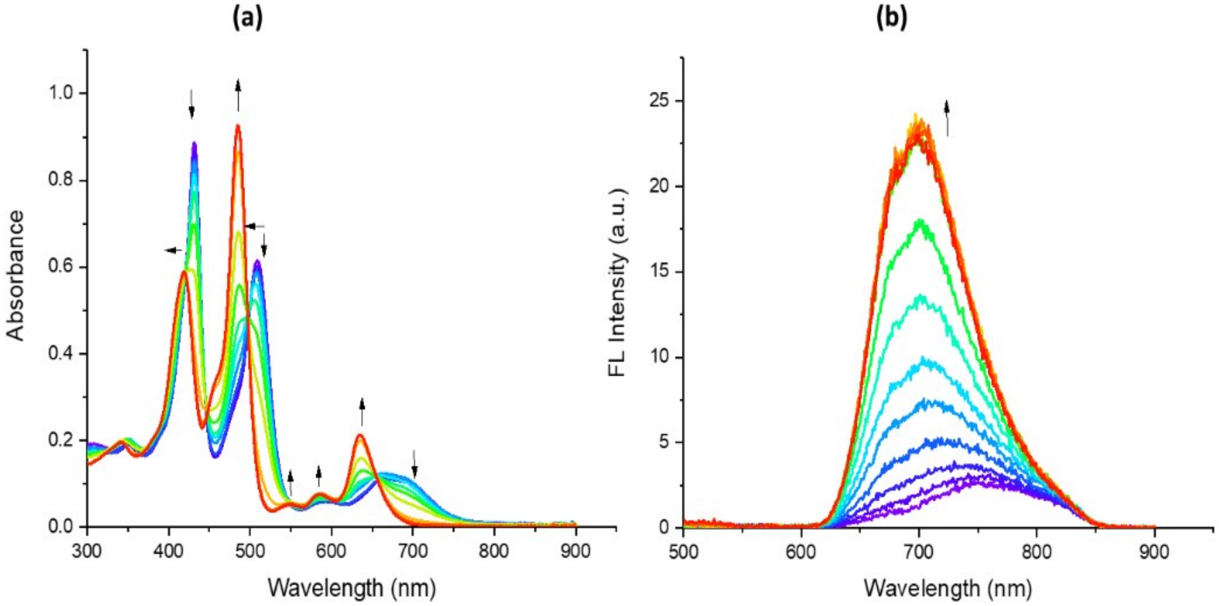

Figure S2. (a) Absorption and (b) fluorescence (excitation = 485 nm) spectral changes observed during addition of sodium tetraphenylborate (2 equivalents each) to a solution of **F^-:ZnP-SQ** (5.5×10^{-6} M) to reveal reversible F^- binding.

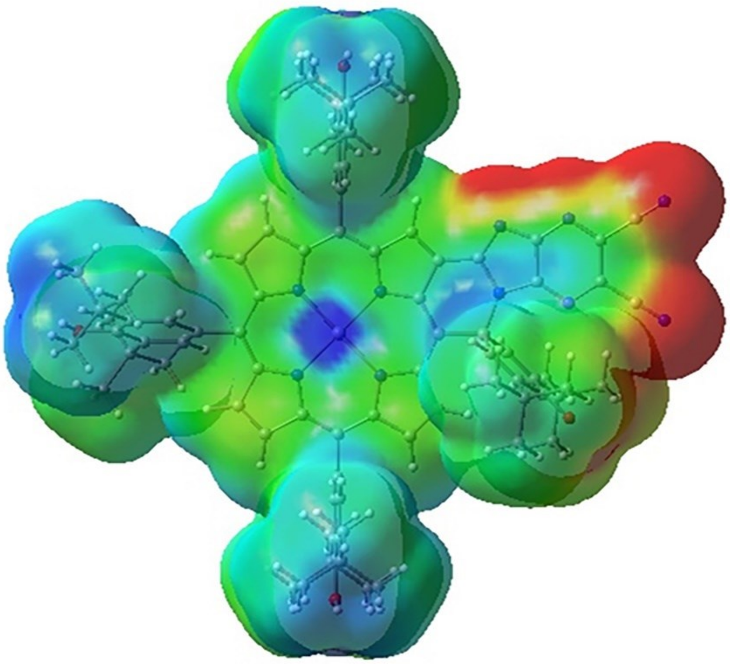

Fig. S3. Molecular electrostatic potential map of **ZnP-SQ**.

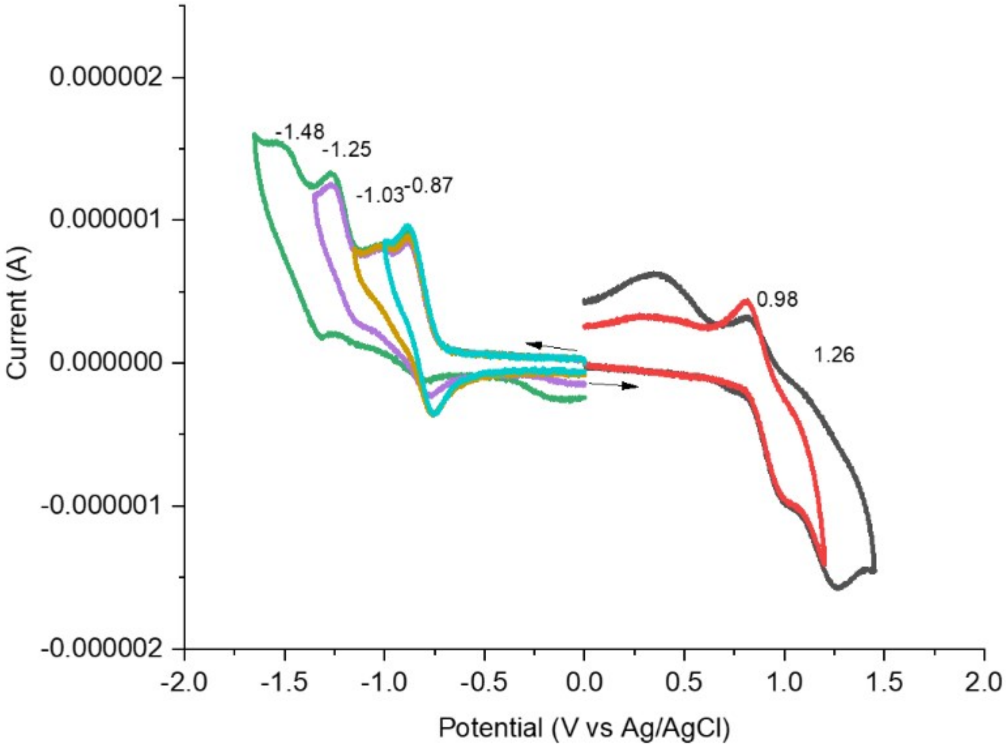

Figure S4. Cyclic voltammograms of **ZnP-SQ** in DCB containing 0.1 M $(TBA)ClO_4$. Scan rate = 600 mV/s on an ice bath.

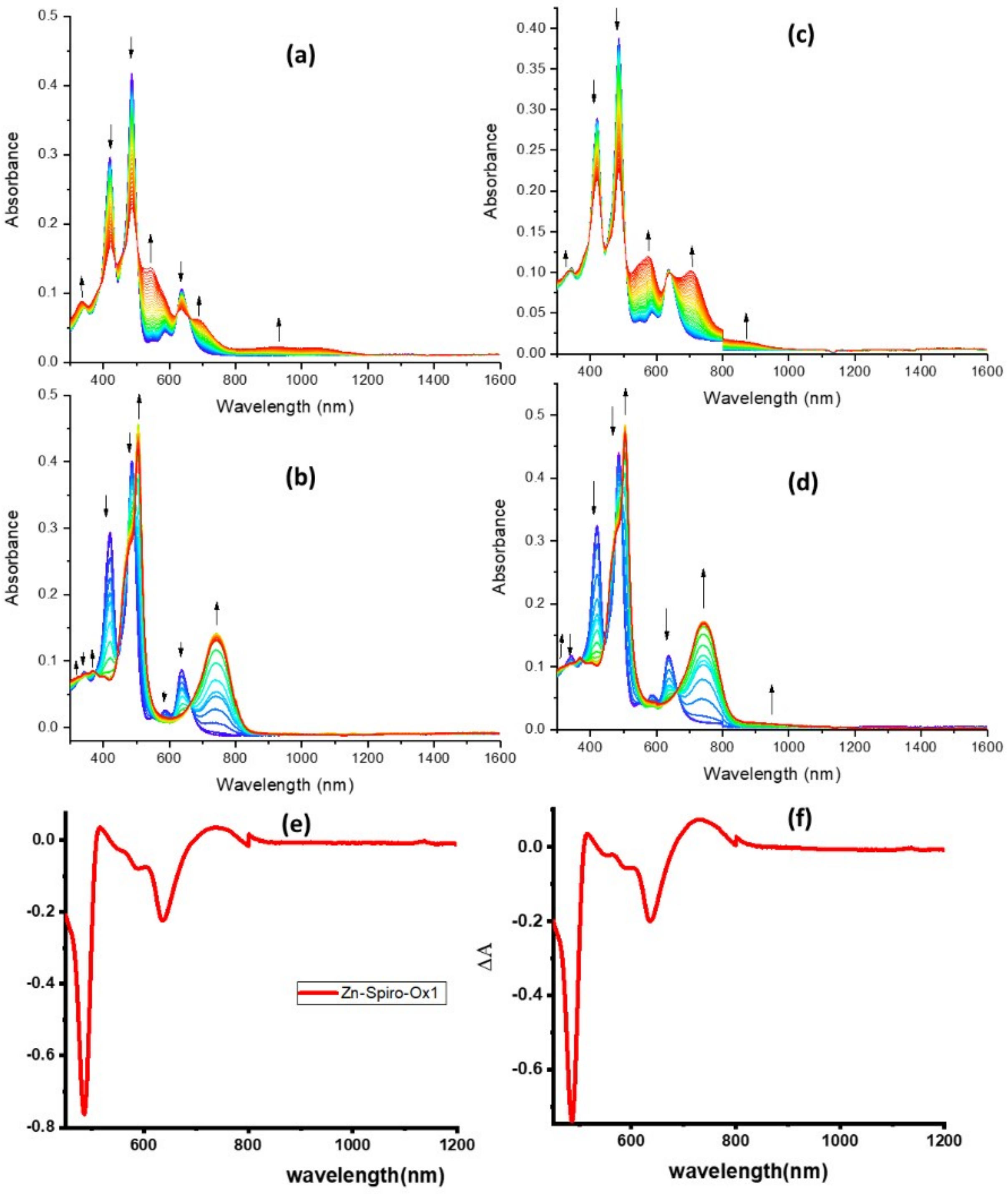

Figure S5. Spectral changes during first oxidation (a and c), and first reduction (b and d) of **ZnP-SQ** (a and b) and **F^-:ZnP-SQ** (c and d) in DCB containing 0.2 M $TBAClO_4$. Figures e and f represent spectrum of the charge separated state of **ZnP-SQ** and **F^-:ZnPSQ**, respectively, deduced from the spectroelectrochemical data

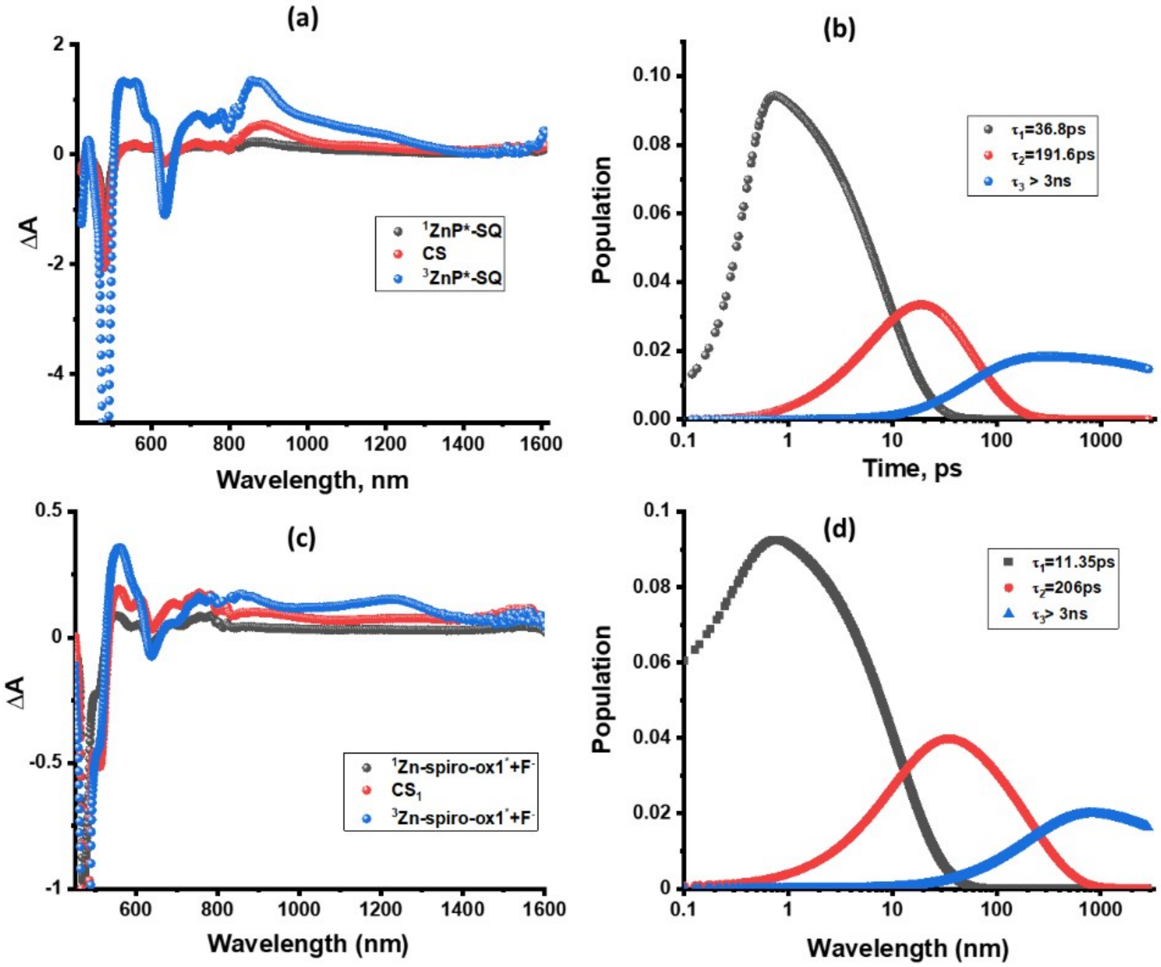

Fig. S6. Species associated spectra (a and c) and population kinetics (b and d) of **ZnP-SQ** (a and b) and **F⁻:ZnP-SQ** (c and d) at the excitation wavlength of 480 nm.

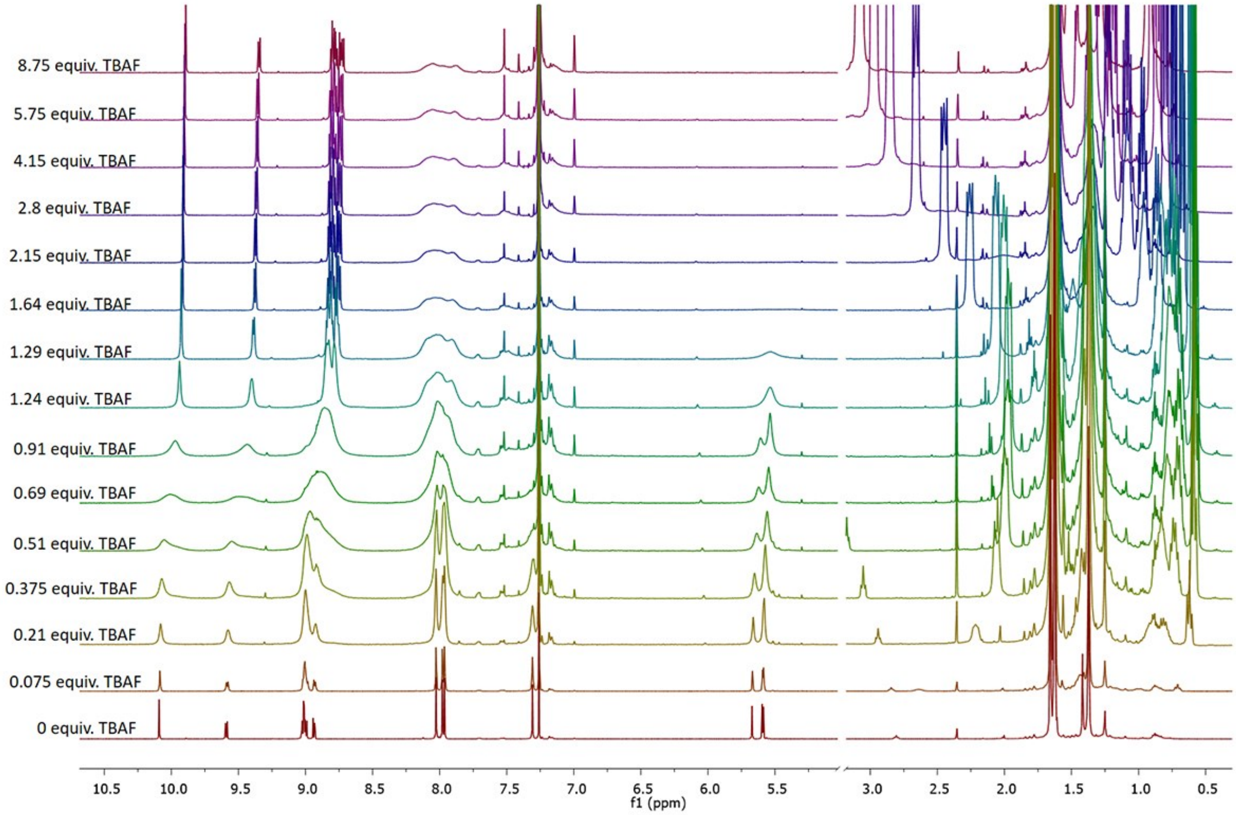

Fig. S7. NMR titration of **ZnP-SQ** with TBAF in $CDCl_3$. Broadening of peaks in the aromatic region up to 1 eq. F^- is due to exchange of fluoride at Zn(II). Above 1 eq., strong binding leads to sharpening of macrocyclic aromatic protons. Meso-substituent proton and OH proton resonances remain broad due to H-bonding at the phenols.

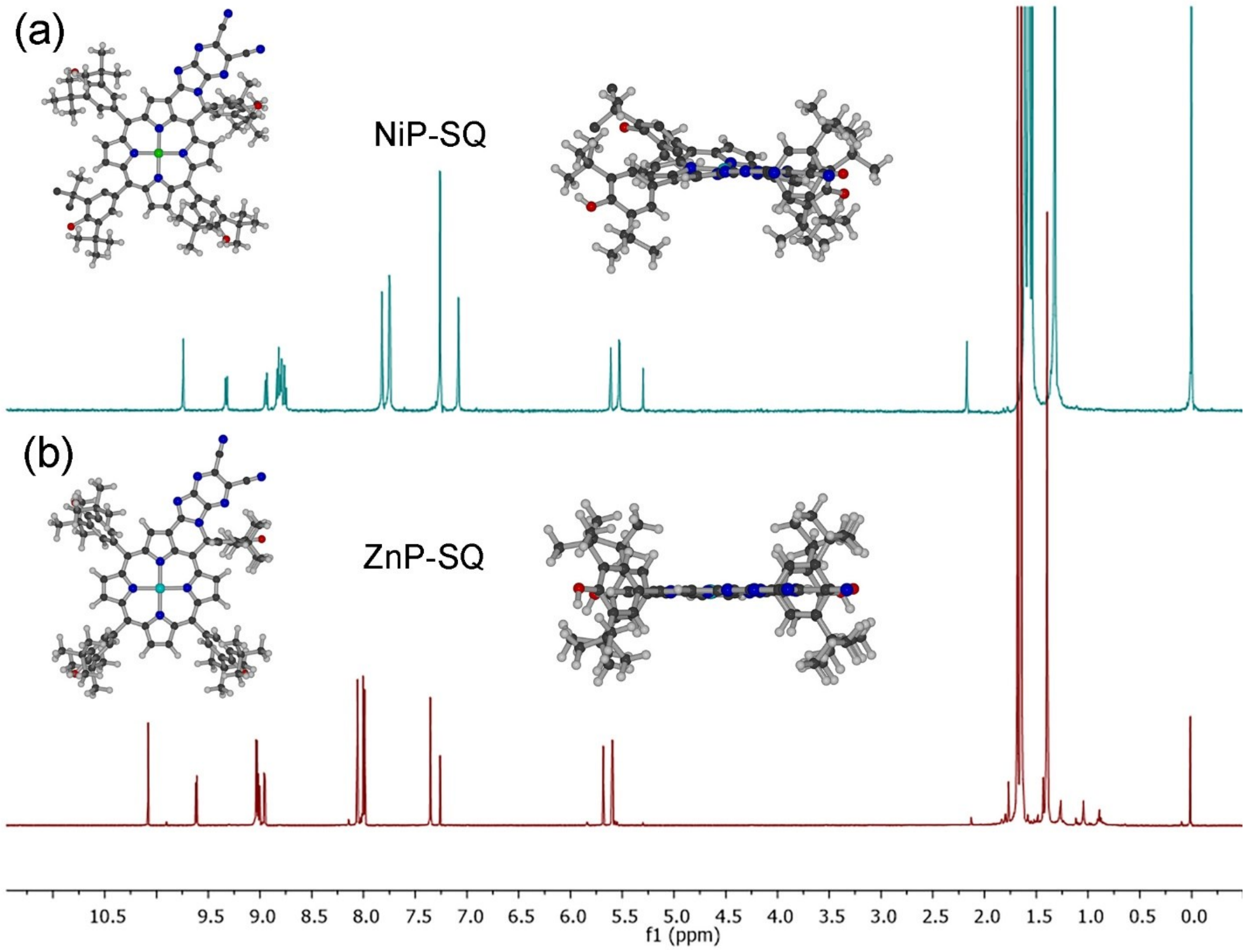

Fig. S8. ^{1}H NMR spectra of (a) **NiP-SQ** (with X-ray crystal structure) and (b) **ZnP-SQ** (with DFT calculated structure). Increased planarity of the macrocycle in **ZnP-SQ** leads to lower field chemical shifts especially of resonances due to aromatic protons in compared to **NiP-SQ**.

4.0 Chemical Analytical Data

^{1}H NMR spectrum of **H_2P-SQ**

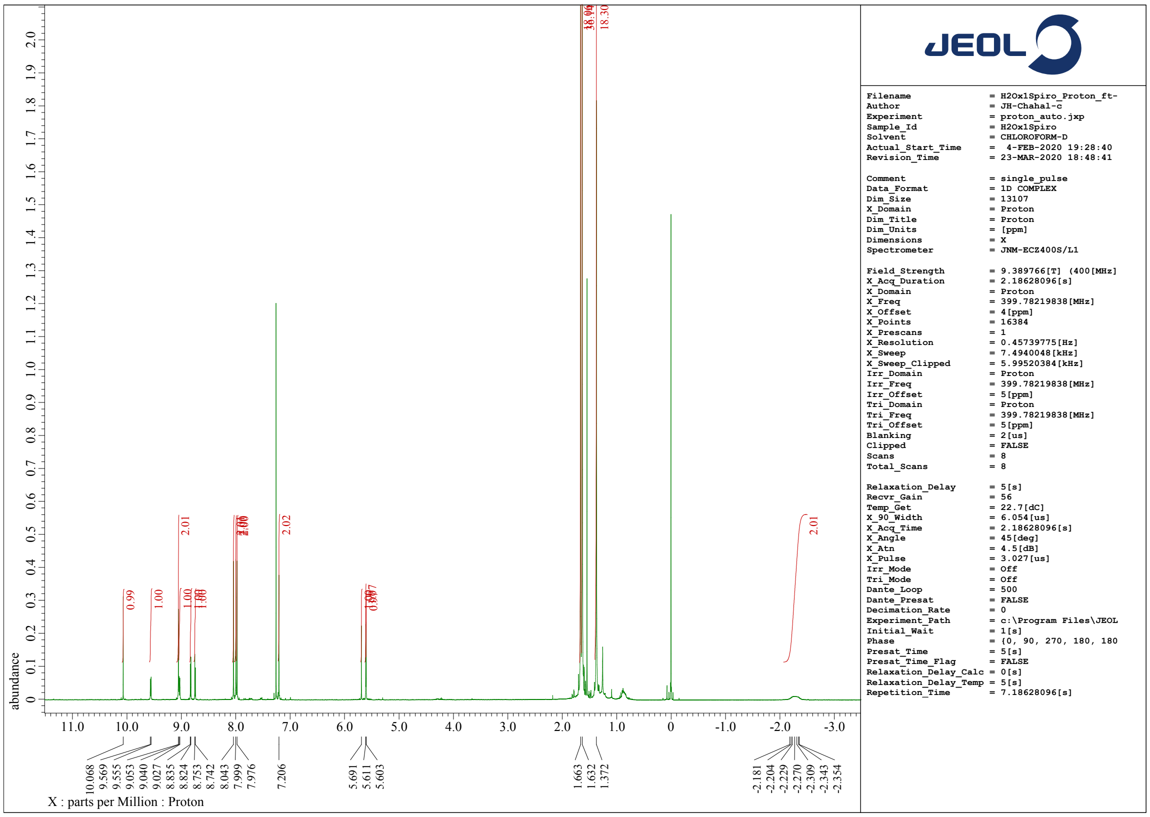

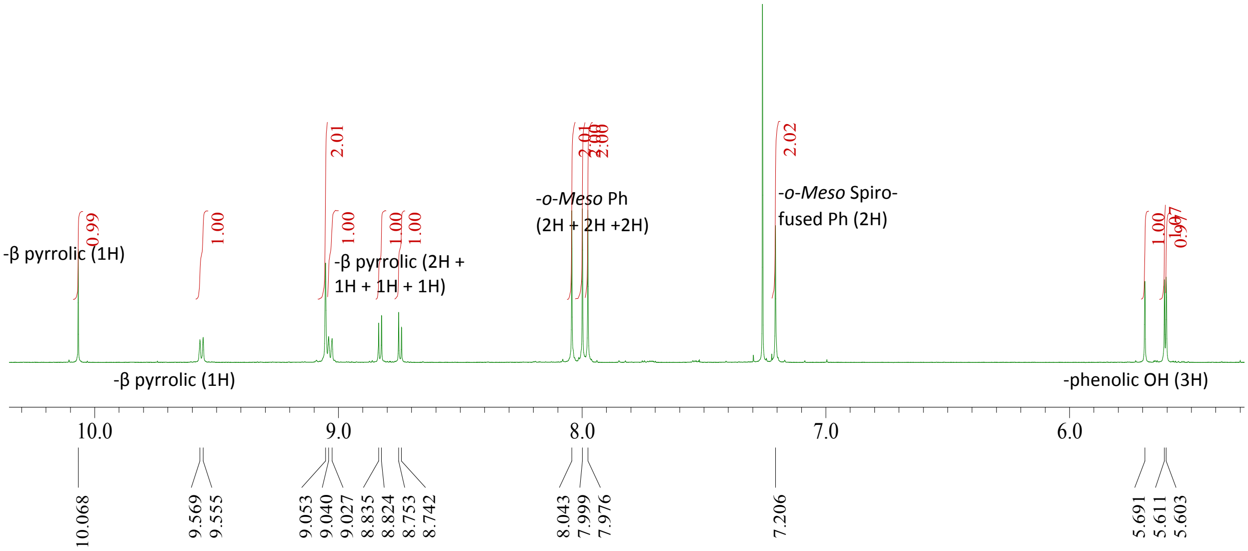

^{13}C NMR spectrum of **H_2P-SQ**

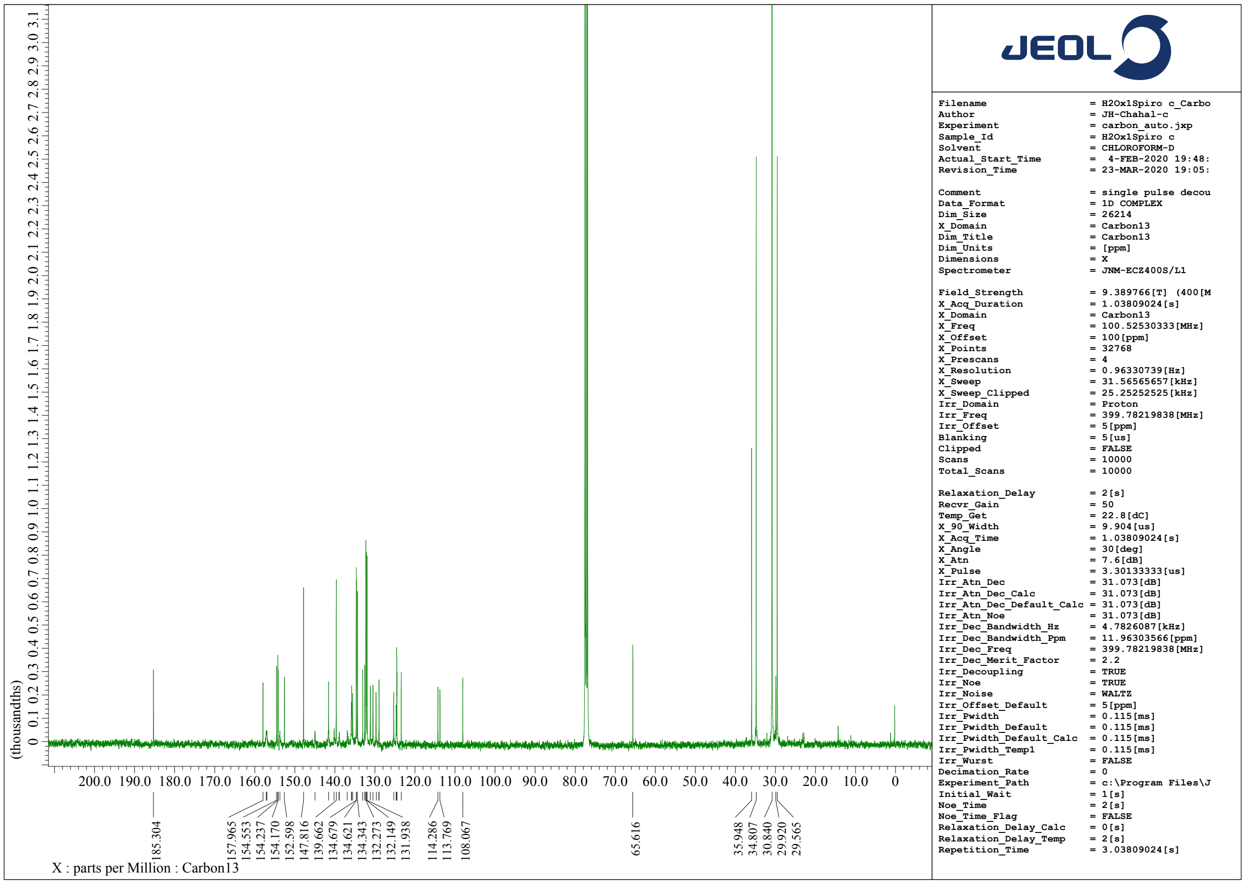

ESI-TOF-MS spectrum of **H_2P-SQ**

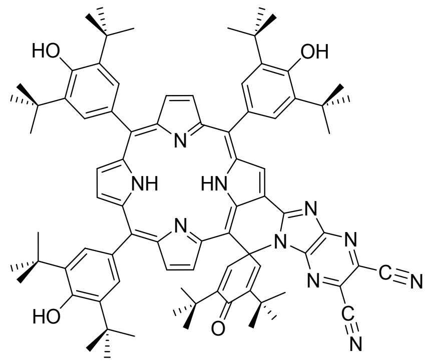

Chemical Formula: $C_{83}H_{92}N_{10}O_4$

Calculated (upper) and experimental (lower) ESI-TOF-MS:

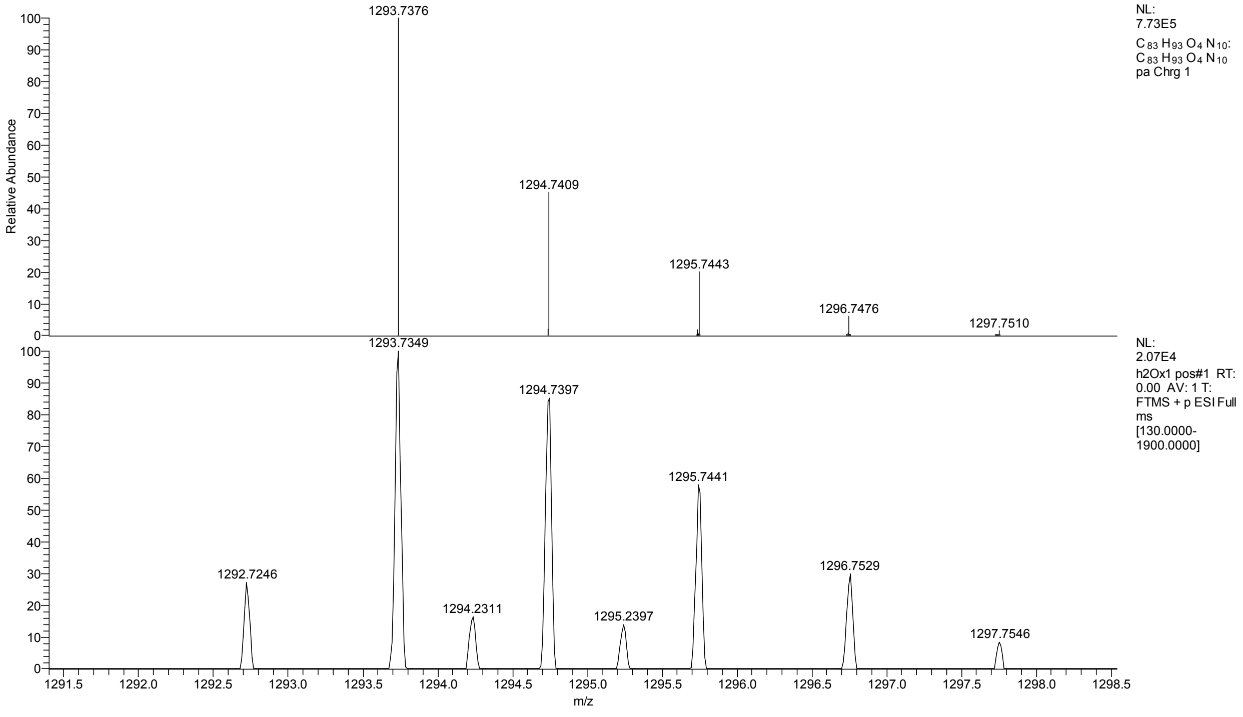

^{1}H NMR spectrum of **ZnP-SQ**

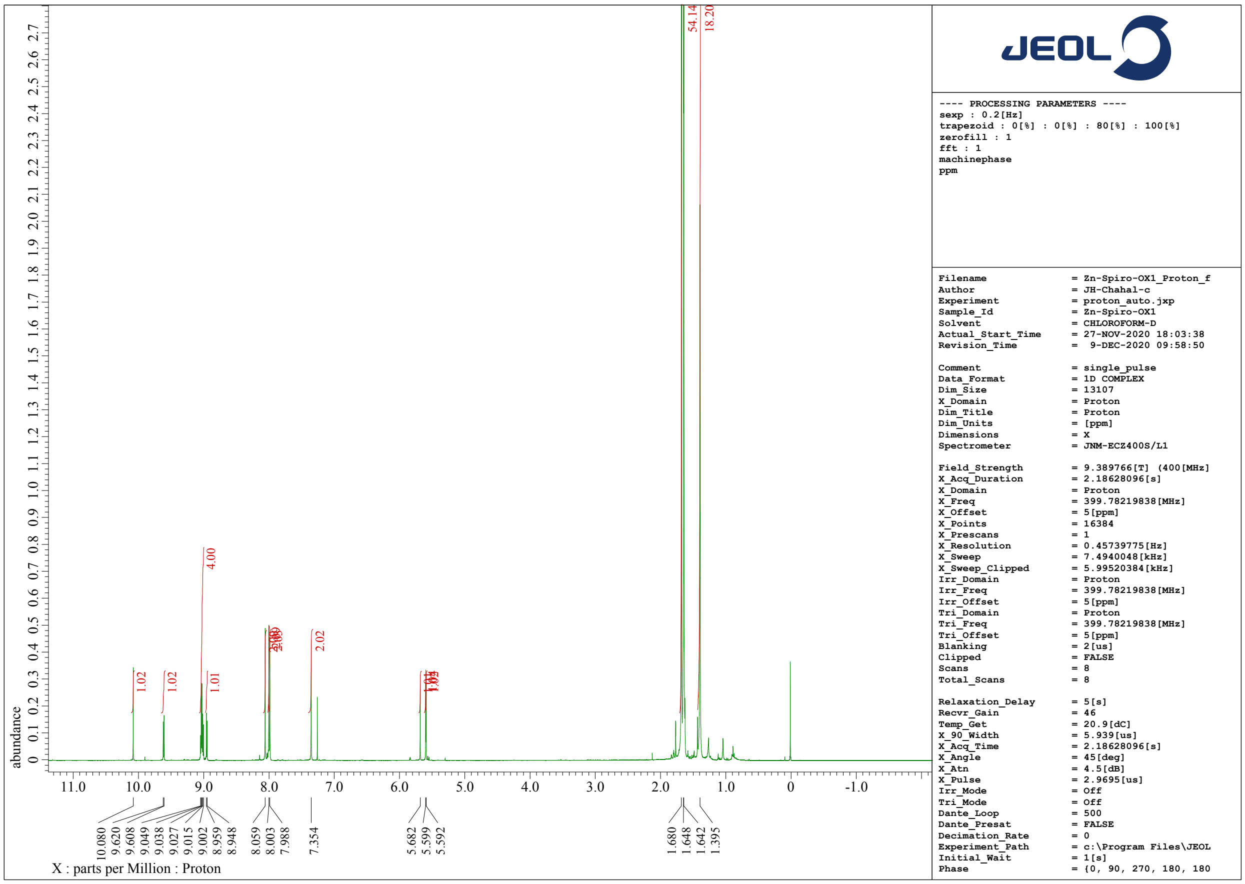

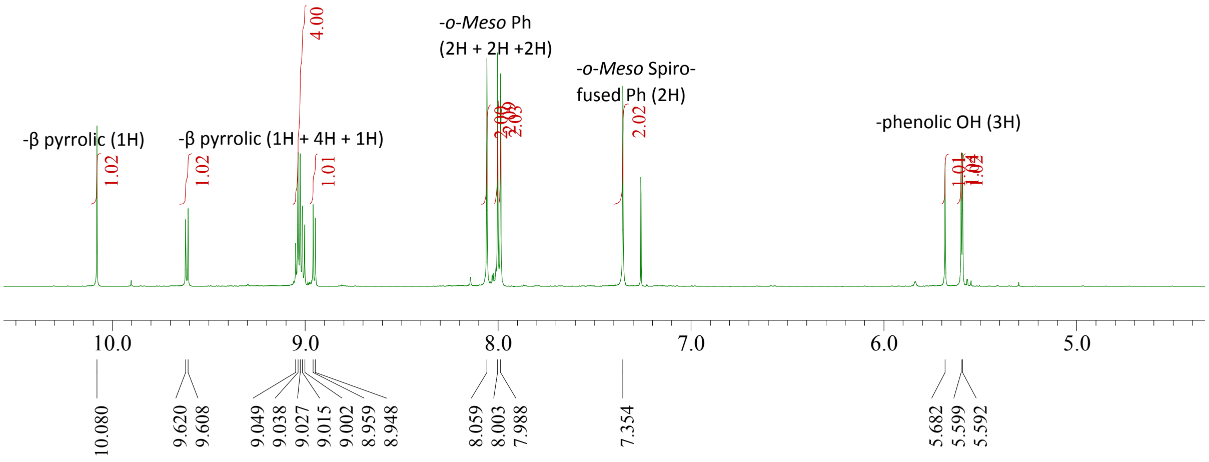

^{13}C NMR spectrum of **ZnP-SQ**

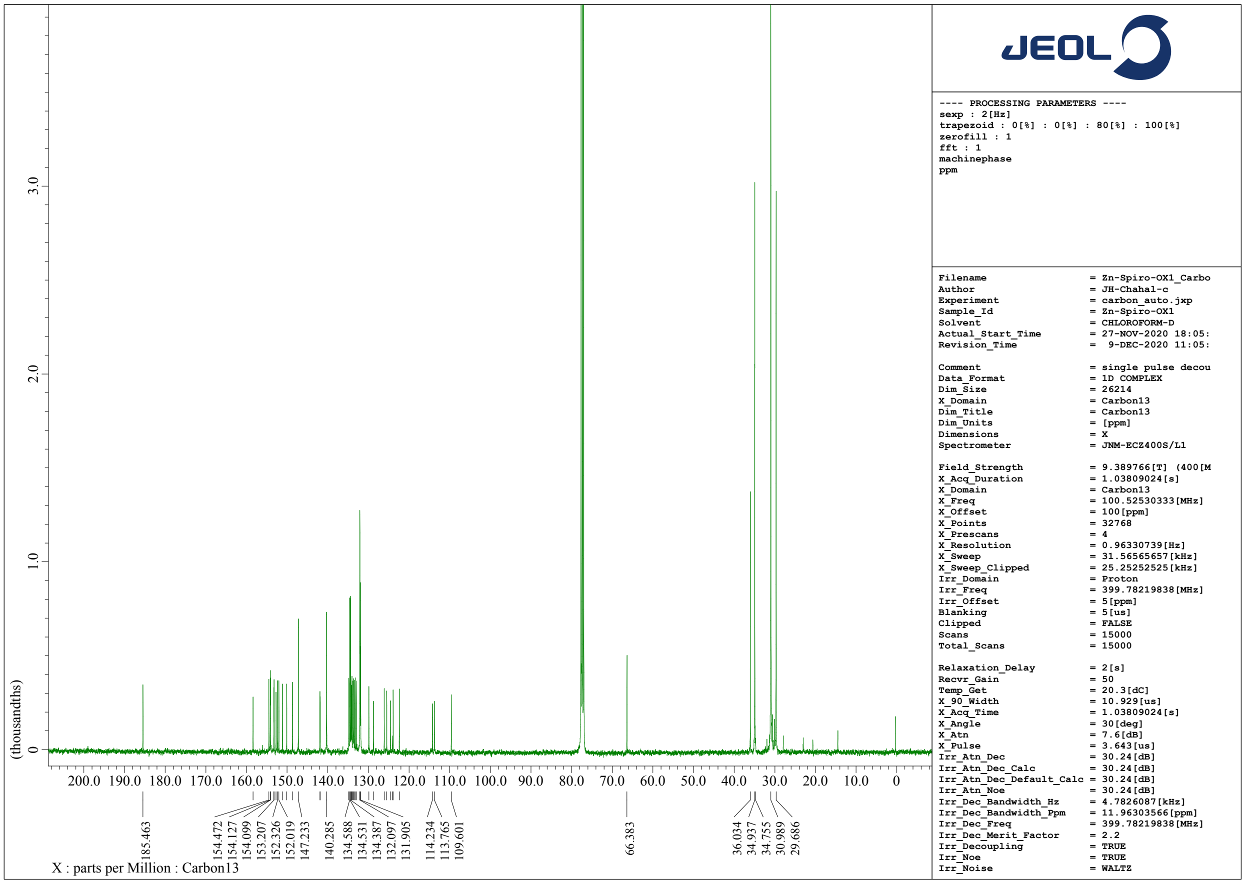

ESI-TOF-MS spectrum of **ZnP-SQ**

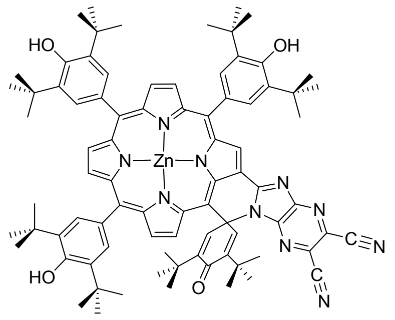

Chemical Formula: $C_{83}H_{90}N_{10}O_4Zn$

Calculated (upper) and experimental (lower) ESI-TOF-MS:

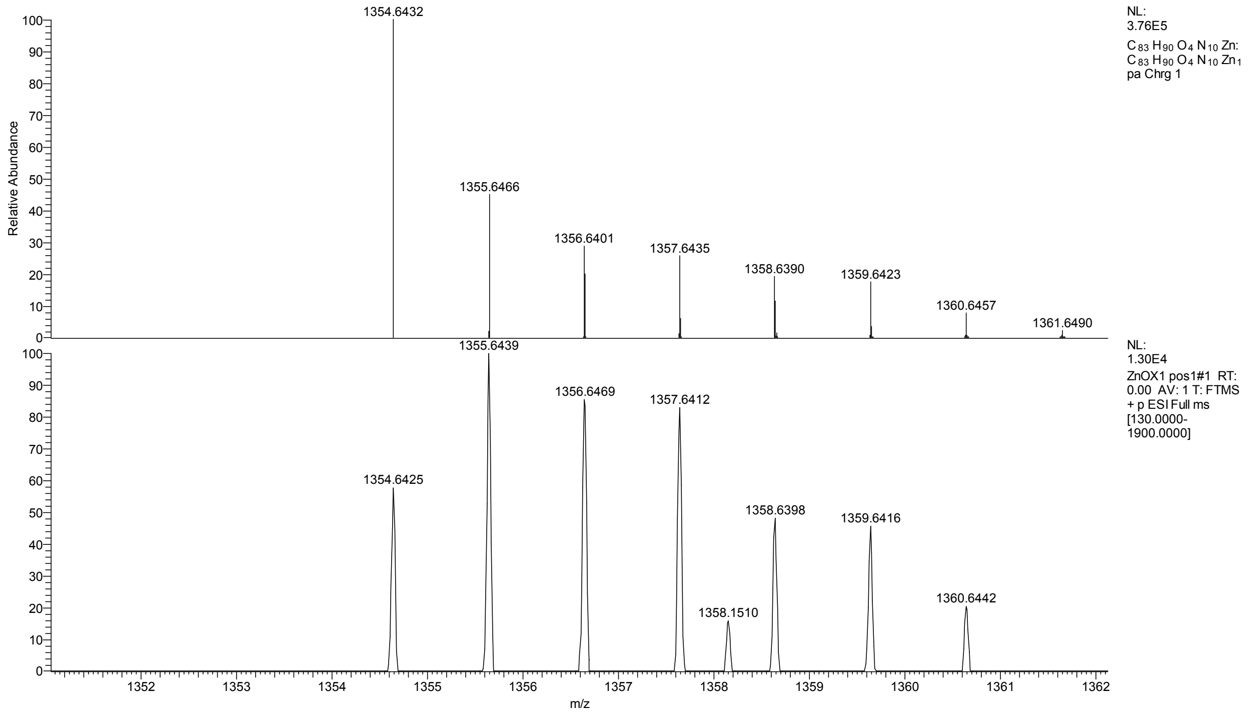

^{1}H NMR spectrum of **F$^-$:ZnP-SQ**

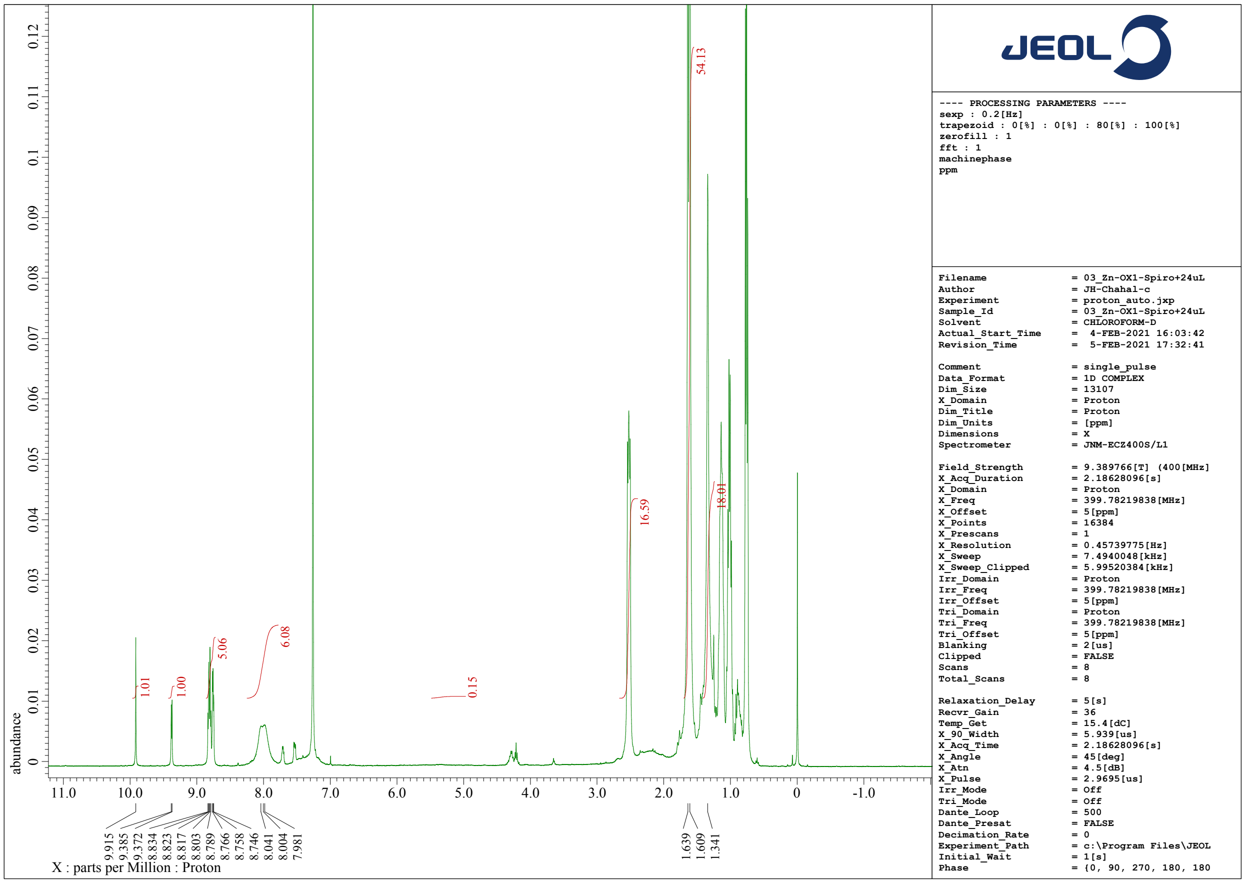

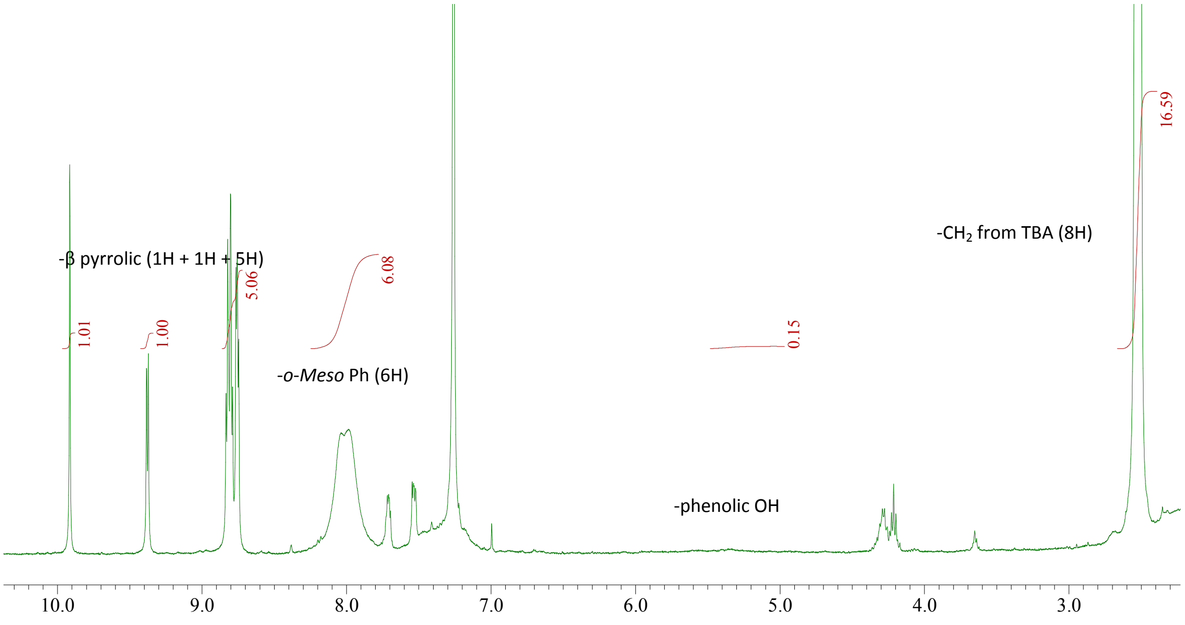

^{13}C NMR spectrum of **F^{-}:ZnP-SQ**

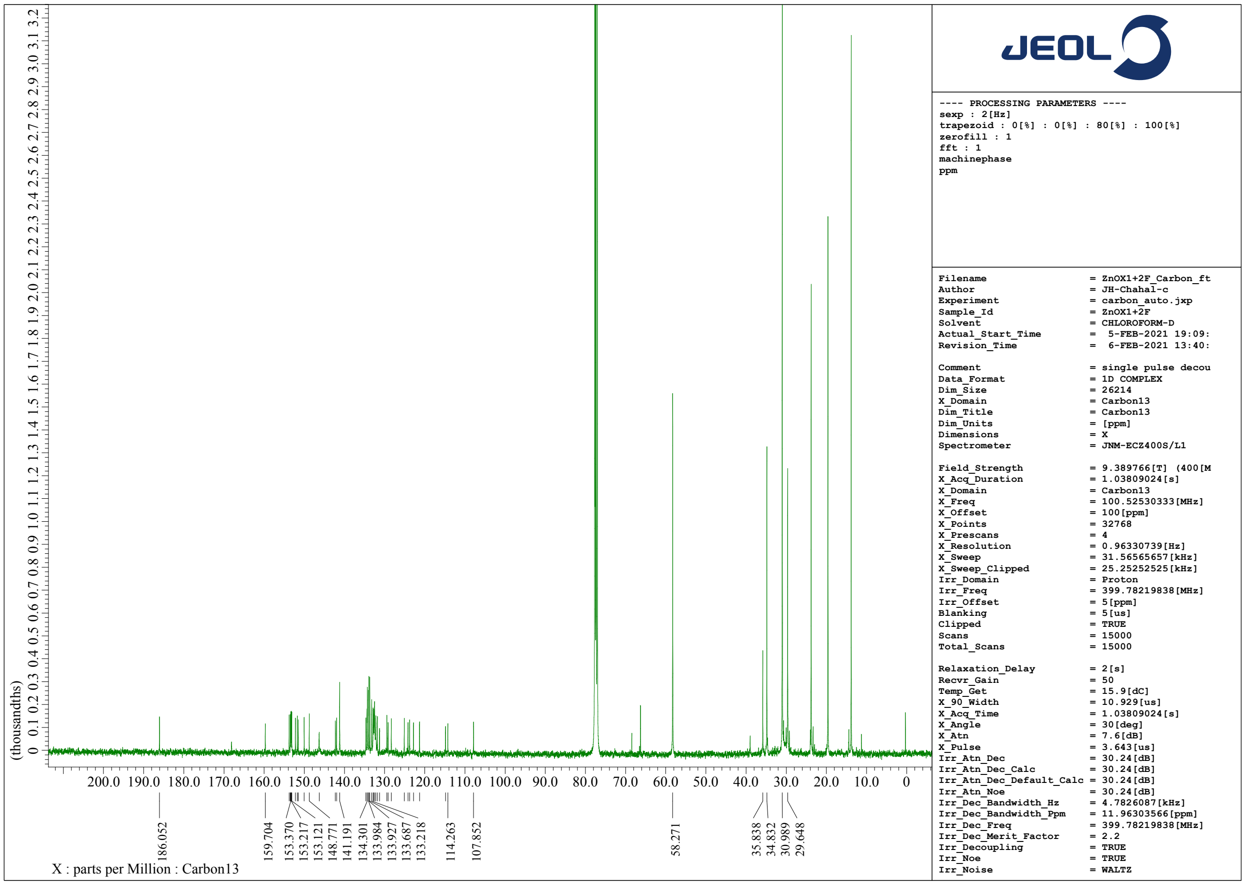

ESI-TOF-MS spectrum of **F⁻:ZnP-SQ**

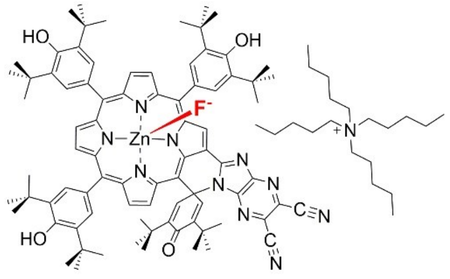

Chemical Formula: $[C_{83}H_{90}N_{10}O_4Zn:F^-][TnBA^+]$

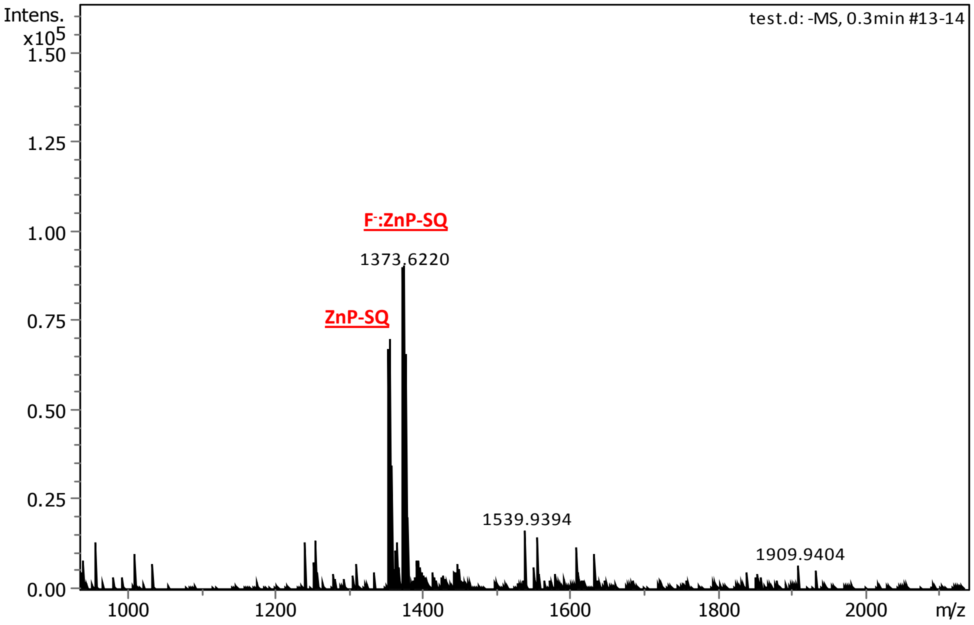

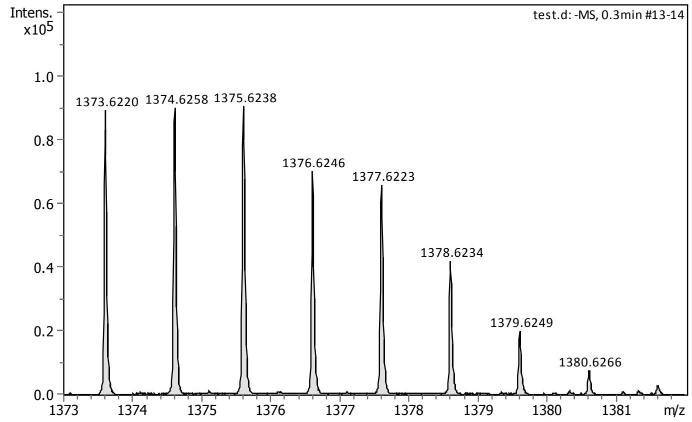

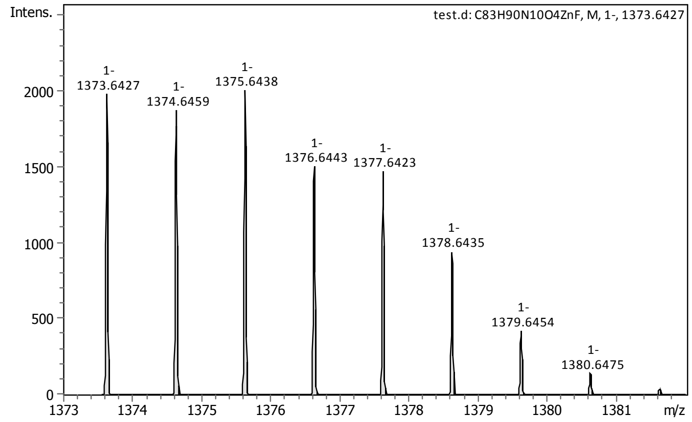

Calculated (upper) and experimental (lower) ESI-TOF-MS of **F^-:ZnP-SQ**:

References

S1. M. K. Chahal, J. Labuta, V. Březina, P. A. Karr, Y. Matsushita, W. A. Webre, D. T. Payne, K. Ariga, F. D'Souza and J. P. Hill, *Dalton Trans.*, 2019, **48**, 15583–15596.
