# Supplementary material for: Anion-enhanced excited state charge separation in a spiro-locked N-heterocycle-fused push-pull zinc porphyrin
Source: Chem Sci. 2021 Feb 24;12(13):4925–30. doi: 10.1039/d1sc00038a (PMC8179616; doi:10.1039/d1sc00038a)
Supplement: SC-012-D1SC00038A-s002 [file SC-012-D1SC00038A-s002.pdf]

Electronic Supplementary Material (ESI) for Chemical Science.
This journal is © The Royal Society of Chemistry 2021

Table of Contents

Anion-enhanced Excited State Charge Separation in a Spiro-locked N-Heterocycle-fused Push-Pull Zinc Porphyrin

Mandeep K. Chahal, Anuradha Liyanage, Ajyal Z. Alsaleh, Paul A. Karr, Jonathan P. Hill* and Francis D'Souza*

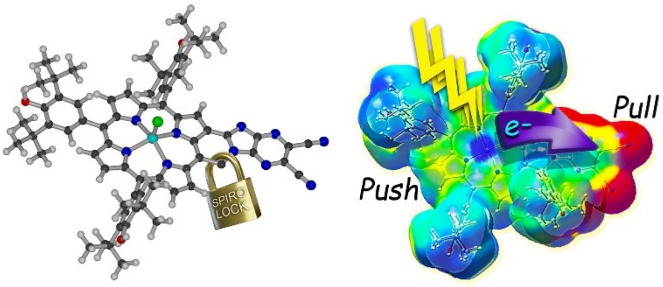

Anion-induced enhancement of excited state charge separation in a spiro-locked nitrogen-heterocycle-fused push-pull zinc porphyrin by ligation of fluoride to the Zn centre is demonstrated using advanced spectroscopic techniques.
